# Tenecteplase versus alteplase in patients with acute ischemic stroke: an updated systematic review and meta-analysis

**DOI:** 10.1186/s40001-025-02983-9

**Published:** 2025-08-08

**Authors:** Abdelmonam M. Hagag, Muhammed E. Kormod, Mahmoud Elmwafy Ads, Mennatullah A. El-Refaay, Omnia M. Abozaid, Omar A. Ghanem, Mazen Yasser, Karim M. Abdelmoaty, Alaa Mahmoud Khedr, Gregory W. Albers

**Affiliations:** 1 Faculty of Medicine, Zagazig University, As Sanafin Al Qibliyyah, Minya Al Qamh, Zagazig, Sharqia Egypt; 2 Faculty of Medicine, Al-Azhar University, Cairo, Egypt; 3 Faculty of Medicine, Mansoura University, Mansoura, Egypt; 4 Faculty of Medicine, October 6 University, Cairo, Egypt; 5 Faculty of Medicine, Minia University, Minia, Egypt; 6 Department of Neurology, Stanford University, Palo Alto, CA USA

**Keywords:** Tenecteplase, Alteplase, Acute ischemic stroke, TNK, RtPA

## Abstract

**Background:**

Stroke was the second leading cause of death and the third leading cause of disability worldwide in 2019. Alteplase is an FDA-approved medication for the treatment of patients with ischemic stroke within 4.5 h. However, Tenecteplase is an alternative. We aimed to assess the safety, efficacy, and the best dose of tenecteplase vs alteplase.

**Methodology:**

We followed the PRISMA 2020 guidelines. We searched PubMed, Scopus, Web of Science, and the Cochrane Library until November 2024. We included RCTs that compared tenecteplase with alteplase in acute ischemic stroke patients within 4.5 h of onset. Relative risk, 95% CI, and random effects models were used. Our primary endpoints were excellent functional outcomes and mortality. An excellent functional outcome was defined as a modified Rankin Score of 0–1 at 90 days. The Risk of Bias 2 tool from Cochrane was used to assess the quality of the studies.

**Results:**

Thirteen RCTs met our inclusion criteria, including 9053 patients, 4416 of whom had tenecteplase. Six studies showed a low risk of bias, and the other 7 had some concerns either in their randomization process or in the deviation from the intended intervention. Tenecteplase 0.25 mg/kg had a significantly better performance in achieving excellent functional outcomes (p value 0.008), but no difference was observed in mortality rates (P value 0.37). On the other hand, no difference was observed in comparing either TNK 0.1 mg/kg or TNK 0.4 mg/kg versus alteplase in the reported outcomes. A meta-regression revealed that a reduction in the baseline NIHSS score had a significant effect on favorable functional outcomes (P value 0.025). Sensitivity analysis for outcomes comparing either TNK 0.25 mg/kg and TNK 0.1 mg/kg versus alteplase showed that no single study removal affected the significance of the study, while Kvistad 2022 and Logallo 2017 removal changed the significance of outcomes comparing TNK 0.4 mg/kg versus alteplase.

**Conclusion:**

When compared with alteplase, 0.25 mg/kg tenecteplase is superior for achieving excellent functional outcomes and is not inferior in terms of major neurological improvement or death rates. TNKs at 0.25 mg/kg appear to be the optimal dose and a desirable alternative to alteplase.

**PROSPERO Registration Number:**

CRD42024596896

**Supplementary Information:**

The online version contains supplementary material available at 10.1186/s40001-025-02983-9.

## Introduction

Stroke is a significant global health burden, being the second-leading cause of death and the third-leading cause of disability worldwide in 2019 [1]. Ischemic stroke (IS) is caused by vessel occlusion, leading to diminished blood flow to the brain tissue, resulting in neurological impairment, and is the most common subtype [1, 2]. Rapid reperfusion via intravenous fibrinolytics with or without mechanical thrombectomy is the main therapeutic goal [3, 4].

Alteplase (tPA) is a tissue plasminogen activator that is used for rapid reperfusion. It is the only fibrinolytic agent that is FDA-approved for managing patients with acute ischemic stroke, and its approval is limited to treatment within 3 h of stroke onset [5]. However, the benefit was extended to 4.5 h on the basis of the European Cooperative Acute Stroke Study (ECASS) [6]. As a result, the American Heart Association/American Stroke Association (AHA/ASA) and the European Stroke Organization (ESO) recommend alteplase as the first-line therapy for AIS for up to 4.5 h [3, 7] On the other hand, alteplase has several limitations, including a short half-life requiring a bolus dose followed by a 1 h infusion, hemorrhagic complications, and recanalization rates below 40% [8–10]. Therefore, exploring alternative therapies is essential.

When compared with alteplase, tenecteplase (TNK) is a third-generation tissue plasminogen activator that is genetically modified with 15-fold greater fibrin specificity and 80-fold greater resistance to plasminogen activator inhibitor-1 [7, 11] which makes it a promising alternative. The FDA first approved TNK for managing patients with ST-segment elevation myocardial infarction (STEMI) before percutaneous coronary intervention (PCI) became the gold standard of management. When compared alteplase, TNK has a longer half-life and lower plasma clearance, allowing its administration as a bolus dose [7, 12]. Several randomized controlled trials (RCTs) have assessed the safety and efficacy of tenecteplase versus alteplase with various TNK doses (0.1, 0.25, and 0.4 mg/kg) for patients with acute ischemic stroke [13–16]. Some national guidelines recommend the use of tenecteplase in ischemic stroke management [17], and the last European Stroke Organization (ESO) guidelines recommend TNK at a dose of 0.25 mg/kg/kg as an alternative to alteplase [18, 19]. Recent RCTs, such as Parsons 2024, Meng 2024, and Muir 2024 [20–22], have provided additional data. This study aimed to evaluate the safety and efficacy of tenecteplase compared with those of alteplase, discuss its limitations, and clarify the optimal dose of tenecteplase. We further aimed to assess the effect of the baseline NIH stroke scale (NIHSS) score, mean age, sex, and hypertension on the efficacy of tenecteplase.

## Methodology

This systematic review and meta-analysis followed the Preferred Reporting Items for Systematic Review and Meta-Analysis 2020 (PRISMA) guidelines [23]. The prespecified protocol was registered on the International Prospective Register of Systematic Reviews (PROSPERO) under the number: CRD42024596896.

We included studies that included the following: (1) patients with acute ischemic stroke within 4.5 h of onset and eligible for thrombolytics (population), (2) those that compared tenecteplase (intervention) with alteplase (comparison), (3) those reporting their safety and efficacy outcomes, including excellent functional outcomes and mortality rates, (outcomes). (4) studies should be randomized control trials (study design), 5/written in the English language, and 6/working with only human patients. We excluded studies that did not meet these criteria, and conference abstract articles, protocols, animal studies, and observational studies.

### Search strategy

We systematically searched four key databases: PubMed, Web of Science, Scopus, and the Cochrane Library, up to 17th November 2024. We conducted a comprehensive search of titles and abstracts via specific keywords: (Tenecteplase OR TNK) AND (Alteplase) AND (Acute ischemic stroke). We used the same search strategy for the four databases, which is provided in File 1 supplementary material.

### Screening and data extraction

After the papers were collected, they were uploaded to EndNote X9 to automatically remove duplicates. The results were then reuploaded to Rayyan for title and abstract screening. Two reviewers (OA and MY) independently screened the papers to identify those suspected of being eligible. Any conflicts were resolved through negotiation with the first author (AM). Four independent reviewers (OA, KM, MA, and ME) then assessed the full articles, dividing the workload among the reviewers. OA and KM independently reviewed half of the articles, whereas MA and ME reviewed the other half. Any conflicts were resolved by revisiting the eligibility criteria and negotiating with the first author (AM).

Four reviewers independently extracted the data, which included the type of study, methods of randomization and blinding, site and ethical approval committee, inclusion criteria for each paper, main results, total sample size, and number of patients in each group. The following additional data were also collected: age, sex, baseline NIHSS score, mRS score, onset-to-needle time, door-to-needle time, risk factors, and study outcomes.

The primary efficacy outcomes were major neurological improvement and excellent functional outcomes. Excellent functional outcomes were defined as mRS scores of 0–1 at 90 days. Major neurological improvement was defined according to the RCT articles. Most studies defined major neurological improvement as a reduction in the NIHSS score of ≥ 8 points at 24 h. Some studies defined it as a reduction of ≥ 4 in some papers, or an NIHSS score of 0 or 1 at 24 h. We summarized the definitions in Table 1. We performed a subgroup analysis to compare studies that used ≥ 8 points at 24 h as a definition and studies that used ≥ 4 points at 24 h to compare the outcome results between the two definitions.

**Table 1:** Summary of the included studies

Study ID				Tenecteplase group		Alteplase group
	Study design	Major neurological improvement definition	Total sample size	Patients N	Dose	Patients N
Haley [16]	Small, multicenter, randomized, double-blind, controlled clinical trial	A ≥ 8-point improvement compared with baseline, or a score of 0, on the National Institutes of Health Stroke Scale5 at 24 h	112	31 Patients (0.1 mg/kg) 31 Patients (0.25 mg/kg) 19 Patients (0.4 g/kg)		31
Parsons [28]	Randomized, open-label, blinded trial	Defined as a reduction from baseline of 8 or more points on the NIHSS	75	25 Patients (0.1 mg/kg) maximum 10 mg 25 Patients (0.25 mg/kg) maximum dose 25 mg		25
Huang [32]	Single-Centre, phase 2, prospective, randomized, open-label, blinded-endpoint study	Reduction of NIHSS score of 8 points or more or NIHSS score of zero or one at 24–48 h post-treatment	104	52	0.25 mg/kg (maximum 25 mg)	52
Logallo [13]	Phase 3, randomized, open-label, blind endpoint, superiority trial	Either an NIHSS score of 0 at 24 h or a reduction in NIHSS score of at least 4 points at 24 h compared with baseline	1100	549	0.4 mg/kg (maximum 40 mg)	551
Campbell 2018	Multicenter, prospective, randomized, open-label, blinded-outcome trial	A reduction of at least 8 points or a score of 0 or 1 on the NIHSS at 72 h, as assessed by site personnel	202	101	0.25 mg/kg of body weight (maximum dose: 25 mg)	101
Menon [29]	The AcT trial was a pragmatic, multicenter, open-label, registry-linked, randomized, controlled trial		1577	806	Tenecteplase 0.25 mg/kg (maximum 25 mg)	771
Li [30]	A multicenter, prospective, randomized, open-label, blinded endpoint, phase II study	Improvement on NIHSS of ≥ 4 points or a score ≤ 1 at day 14	236	60 Patients (0.1 mg/kg) 57 Patients (0.25 mg/kg) 60 Patients (0.32 g/kg) To a maximum dose 40 mg		59
Kvistad [31]	Multicenter, randomized, open-label, blinded endpoint, phase 3 study	A reduction in NIHSS score of at least 4 points at 24 h compared with baseline	204	100	0·4 mg/kg up to a maximum of 40 mg	104
Bivard [14]	Randomized, open-label, blinded endpoint, phase 2 trial		104	55	(0·25 mg/kg [maximum 25 mg]	49
Wang [27]	A phase 3, multicenter, prospective, open-label, blinded-endpoint, randomized controlled, non-inferiority trial	Decrease of at least 4 points, a score no more than 1 at 24 h and at 7 days, or discharge, whichever occurred first	1417	710	(0·25 mg/kg, maximum dose of 25 mg)	707
Parsons [22]	A phase 3, multicenter, randomized, prospective, open-label, masked endpoint, noninferiority clinical trial	A reduction of at least 8 points or a score of 0 or 1 on the National Institutes of Health Stroke Scale (NIHSS) at 24 h	680	339	0·25 mg/kg bodyweight (maximum dose of 25 mg)	341
Meng [21]	A multicenter, active-controlled, parallel group, randomized, open-label, blinded end point, phase 3 noninferiority study	NIHSS score of 0 or at least a 4-point improvement from baseline	1465	732	0.25 mg/kg; maximum dose, 25 mg	733
Muir [20]	Randomized, parallel group, open-label trial	An improvement of 8 or more points or a return to a total score of 0 or 1 points on the NIHSS	1777	885	0.25 mg/kg; maximum dose, 25 mg	892

^*^Standard alteplase dose is0.9 mg/kg (maximum 90 mg), 10% as an initial bolus followed by infusion over 1 h

The primary safety outcomes were symptomatic intracranial hemorrhage (sICH) within 24, 36, and 48 h, as well as all mortality rates at 3 months. Symptomatic intracranial hemorrhage was defined according to the European Cooperative Acute Stroke Study III criteria (ECASS III) as any hemorrhage associated with neurological deterioration, indicated by an increase in the National Institutes of Health Stroke Scale (NIHSS) score of at least 4 points or more, or death, with hemorrhage being identified as the predominant cause of death or worsening [24].

Other outcomes included favorable functional outcomes, defined as mRS scores of 0–2 at 3 months; poor functional outcomes, defined as mRS scores of 5–6 at 3 months; and any intracranial hemorrhage at 24 and 48 h, defined as any hemorrhage transformation or parenchymal hematoma, according to the European Cooperative Acute Stroke Study I criteria (ECASS I) [6].

### Quality assessment and statistical analysis

Since all the studies were RCTs, two independent reviewers (AM and MA) used the Risk of Bias 2 (RoB 2) tool [25] developed by the Cochrane Collaboration, to assess the risk of bias. Any conflicts between the reviewers were resolved by consensus.

We used Review Manager software (RevMan) version 5.4 and comprehensive meta-analysis (CMA) version 3.7z to conduct the meta-analysis. We used the Risk ratio (RR) with a 95% confidence interval (95% CI) to perform Mantel–Haenszel effect size weighting studies. A random effects model by DerSimonian and Laird [26] was used to calculate pooled estimates due to variation in the study setting and the inclusion criteria. Interstudy heterogeneity was measured by I^2^ and Cochran's Q test values, with a Cochran's Q p value ≤ 0.1 considered significant for heterogeneity. I^2^ values of 25%, 50%, and 75% were considered low, moderate, and high degrees of heterogeneity, respectively.

A leave-one-out test was used to address heterogeneity and assess the effectiveness of removing a single study on the significance of the outcome.

Subgroup analysis was performed on the basis of the tenecteplase group, and we assessed the major neurological improvements in the study setting. In addition, because most studies used 0.25 mg/kg tenecteplase, we further performed a separate analysis for this dose.

Publication bias was assessed via a funnel plot and Egger’s test, with a P value ≤ 0.05 considered significant for publication bias, and any bias was solved via the trim and fill test.

On-model meta-regression was used for outcomes from more than 10 studies to assess the effects of mean age, mean NIHSS scores sex and hypertension on the tenecteplase safety and efficacy profile.

## Results

**Fig. 1:**
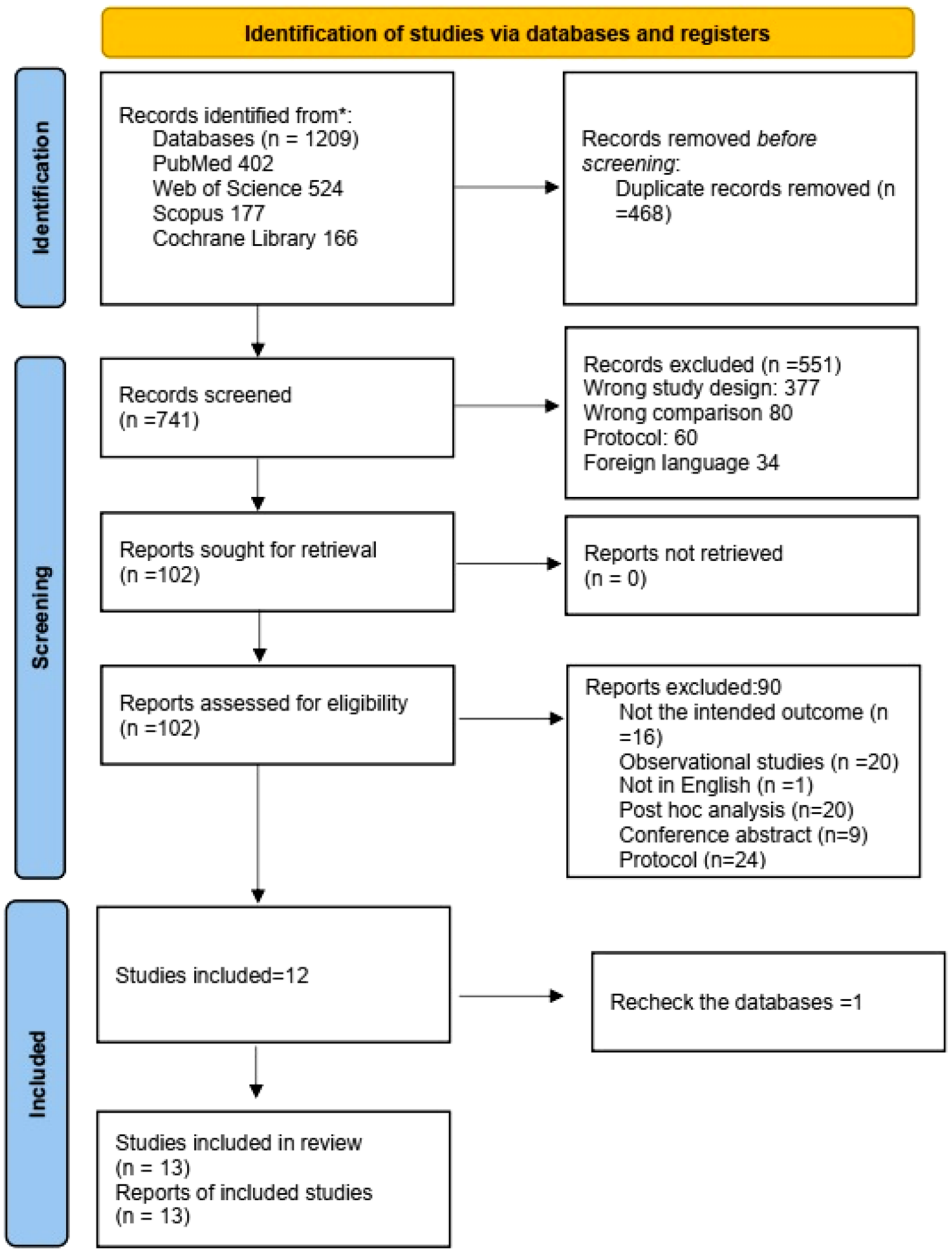
PRISMA flow chart for selecting articles process

We searched the database until 17th November 2024, revealing 1209 publications, which reached 741 after removing duplications. After title and abstract screening, 102 articles were subjected to the full-text screening, resulting in 13 RCTs that met the inclusion criteria [13–16, 20–22, 27–32]. The most notable causes of exclusion were incorrect study designs and protocols. Figure 1 shows the PRISMA 2020 flowchart, which summarizes the selection process.

### Characteristics of the selected articles

Among the 13 RCTs, the total number of patients was 9053, of which 4416 received alteplase and 4637 received Tenecteplase. Table 1 summarizes the included studies. Twelve studies were open-label randomized clinical trials with blind endpoints, whereas Heley 2010 [16] was a double-blinded randomized controlled trial. Studies used different types of randomizations; most of them used block randomization, but a few used stratifications, and a mix of randomization and minimization. The smallest study in terms of sample size was Parsons 2012 [28], which included only 75 patients, and the largest was Muir 2024 [20], which included 1777 patients. Most studies have tested 0.25 mg/kg tenecteplase with a maximum dose of 25 mg [14–16, 20–22, 27–30, 32]; however, some studies have tested 0.4 mg/kg TNK [13, 16, 31], and others have tested 0.1 mg/kg [16, 28, 30] and 0.32 mg/kg TNK [30]. The inclusion criteria and summary of the results are provided in Table S1 in the supplemental materials.

Table 2 summarizes the characteristics of the patients included. The overall mean and standard deviation of the age were 69.72 ± 13.4 and for the tenecteplase group and 69.31 ± 13.4 for the alteplase group. The median and interquartile range (IQR) of the baseline NIHSS score were also very similar between the intervention and control groups. In addition, the prestroke modified Rankin scores were typically between 0 and 1. The most common risk factors were hypertension and lipid disorders.

**Table 2:** Baseline characteristics

Study ID	Study group				Premorbid modified Rankin Scale score (mRS)						Risk factors				
		Age	Sex (Males)	Baseline NIHSS score	0	1	2	≥ 3	Onset-to-needle time (min)	Door-to-needle time (min)	HTN	Diabetes	Hyperlipidemia/cholesterolemia	Atrial fibrillation/Arrhythmia	Smoking
		Mean (SD)	N (%)	Median (IQR)	N (%)	N (%)	N (%)	N (%)	Median (IQR)	Median (IQR)	N (%)	N (%)	N (%)	N (%)	N (%)
Haley [16]	TNK 0.1 mg/kg	67 (19)	12(39%)	8(5–11)	24(77%)		7(23%)				25(81%)	6(19%)	16(52%)		2(6.5%)
	TNK 0.25 mg/kg	69(15)	16(52%)	10(6–15)	28(90%)		3(10%)				25(81%)	7(23%)	15(48%)		7(23%)
	TNK 0.4 mg/kg	68 (16)	13(68%)	9(5–17)	19(100%)		0				17(90%)	4(21%)	8(42%)		0
	rtPA 0.9 mg/kg	72(16)	17(51%)	13(5–17)	26(84%)		5(16%)				22(71%)	4(13%)	17(55%)		7(23%)
Parsons [28]	TNK 0.1 mg/kg	72(6.9)	13(52%)	14.5(3.105)							16(24%)	8(32%)	13(52%)	9(36%)	9(36%)
	TNK.25 mg/kg	68(9.4)	13(52%)	14.6(3.105)							16(24%)	6(24%)	15(60%)	13(52%)	5(20%)
	rtPA	70(8.4)	12(48%)	14(3.105)							15(60%)	1(4%)	9(36%)	6(24%)	1(4%)
Huang [32]	TNK 0.25 mg/kg	71(13)	30(64%)	12(9–18)					180(156–215)	42(22.95)	20(43%)	7(15%)	4(9%)	19(40%)	13(28%)
	rtPA	71(12)	31(63%)	11(8–16)					200(160–220)	38(25.65)	28(57%)	7(14%)	7(14%)	15(31%)	10(20%)
Logallo [13]	TNK 0.4 mg/kg	70.8(14.4)	321(58%)	4(2–7)	435(79%)	62(11%)	25(5%)	27(5%)	118(79–180)	32(22–47)	246(45%)	72(13%)	61(11%)	50(9%)	169(31%)
	rtPA	71.2(13.2)	339(62%)	4(2–8)	425(77%)	65(12%)	26(5%)	35(6%)	111(80–174)	34(25–50)	236(43%)	74(13%)	65(12%)	69(13%)	177(32%)
Campbell [15]	TNK 0.25 mg/kg	70.4(15.1)	58(57%)	17(12–22)	76(75%)	15(15%)	3(3%)	7(7%)	125(102–156)		64(63%)	10(10%)		27(27%)	18(18%)
	rtPA	71.9(13.7)	52(51%)	17(12–22)	81(80%)	6(6%)	2(2%)	12(12%)	134(104–176)		63(62%)	18(18%)		40(40%)	11(11%)
Menon [29]	TNK	74(14.81)	424(52.6%)	9(6–16)					128(93–186)	36(27–49)					
	rtPA	73(14.81)	398(51.6%)	10(6–17)					131(95–188)	37(29–52)					
Li [30]	TNK 0.1 mg/kg	62.4(11.1)	48(80%)	7(5.0–10.0)	55(91.7%)	5(8.3%)	0		154(56–195)	71(28–149)	43(71.7%)	14(23.3%)	17(28.3%)	8(13.3%)	25(41.7%)
	TNK 0.25 mg/kg	64.3(12.8)	42(73.7%)	8(5.0–12.0)	54(94.7%)	3(5.3%)	0		149(80–179)	60(5–180)	37(64.9%)	9(15.8%)	13(22.8%)	4(7%)	25(43.9%)
	TNK 0.32 mg/kg	64.8(12.1)	42(70%)	7.5(6.0–12.0)	47(78.3%)	11(18.3%)	2(3.3%)		147(69–220)	69(10–134)	35(58.3%)	15(25%)	10(16.7%)	14(23.3%)	21(35%)
	rtPA	66.5(12.6)	38(64.4%)	8(5.0–12.0)	50(84.8%)	5(8.5%)	4(6.8%)		153(18–187)	71(10–146)	42(71.2%)	11(18.6%)	11(18.6%)	6(10.2%)	24(40.7%)
Kvistad [31]	TNK 0.4 mg/kg	73·2(12·6)	45(45%)	11·5(8–17)	60(60·0%)	25(25%)	11(11%)	4(4%)	92·5(74–143)		56(56%)	17(17%)	30(30%)	9(9%)	24(24%)
	rtPA	68·6(15·6)	53(51%)	11(8–17·5)	76(73·1%)	19(18.3%)	7(6.7%)	2(1.9%)	99(73–143)		48(46%)	11(11%)	33(32%)	8(8%)	25(24%)
Bivard [14]	TNK0.25 mg/kg	73.33(18.27)	33(60%)	8(5–14)	47(85%)	4(7%)	1(2%)	3(5%)	97(68–157)	30(25–38)	30(55%)	11(30%)	21(38%)	8,54(15%)	8,54(15%)
	rtPA	71.33(14.51)	30(61%)	8(5–17)	39(80%)	1(2%)	3(6%)	6(12%)	92(66–31)	37(32–43)	31(63%)	17/48(35%)	22(45%)	7,48(15%)	9(18%)
Wang [27]	TNK0.25 mg/kg	66.00(11.14)	492(69%)	7(5–10)	634(89%)	76(11%)			180(135–222)	58(45–78)	510(72%)	172(24%)	130(18%)	137(19%)	266(38%)
	rtPA	65.00(10.4)	479(68%)	7(6–10)	633(85.5%)	74(10.5%)			178·5(135–230)	61(48–84)	512(72%)	207(29%)	160(23%)	146(21%)	276(39%)
Parsons [22]	TNK0.25 mg/kg	74(12.66)	202(60%)	7(4–11)	285(84%)	54(16%)			151(120–189)	65(48–86)	218(64%)	58(19.7%)	122(41.4%)	62(18%)	50(16.9%)
	rtPA	72.33(13.4)	218(64%)	7(5–10)	297(87%)	44(13%)			155(125–199)	64(50–85)	210(62%)	57(18.7%)	116(38%)	66(19%)	47(15.4%)
Meng [21]	TNK0.25 mg/kg	65.67(11.14)	517(70.6%)	6(5.0–8.5)								199(27.2%)		108(14.8%)	
	rtPA	65(11.88)	502(68.5%)	6(5.0–9.0)								204(27.8%)		97(13.2%)	
Muir [20]	TNK0.25 mg/kg	70.4(12.5)	533(60%)	7(5–13)	660(75%)	182(21%)	43(5%)		143(115–188)	47(34–62)	461(552%)	157(18%)	245(28%)	95(11%)	180(20%)
	rtPA	70.4(13.4)	527(59%)	7(5–12)	625(70%)	202(23%)	65(7&)		147(113–185)	46(36–58)	466(52%)	141(16%)	259(29%)	92(10%)	170(19%)

### Quality assessment

Six studies [13–15, 28, 30, 32] had a low risk of bias, whereas the remaining 7 had some concerns [16, 20–22, 27, 29, 31]. This appears to be due to randomization errors or deviation from the intended intervention. For Kvistad [31], an observed difference in patients’ baseline characteristics was found between the two groups, and in Haley [16], two patients were not randomized, resulting in an error in the randomization process in both studies. In other studies, few patients were assigned to take one drug but actually received the comparator drug, which led to a deviation from the intended intervention [20–22, 27, 29]. See Fig. 1 supplementary material.

### Study outcomes

#### Efficacy outcomes

**Fig. 2:**
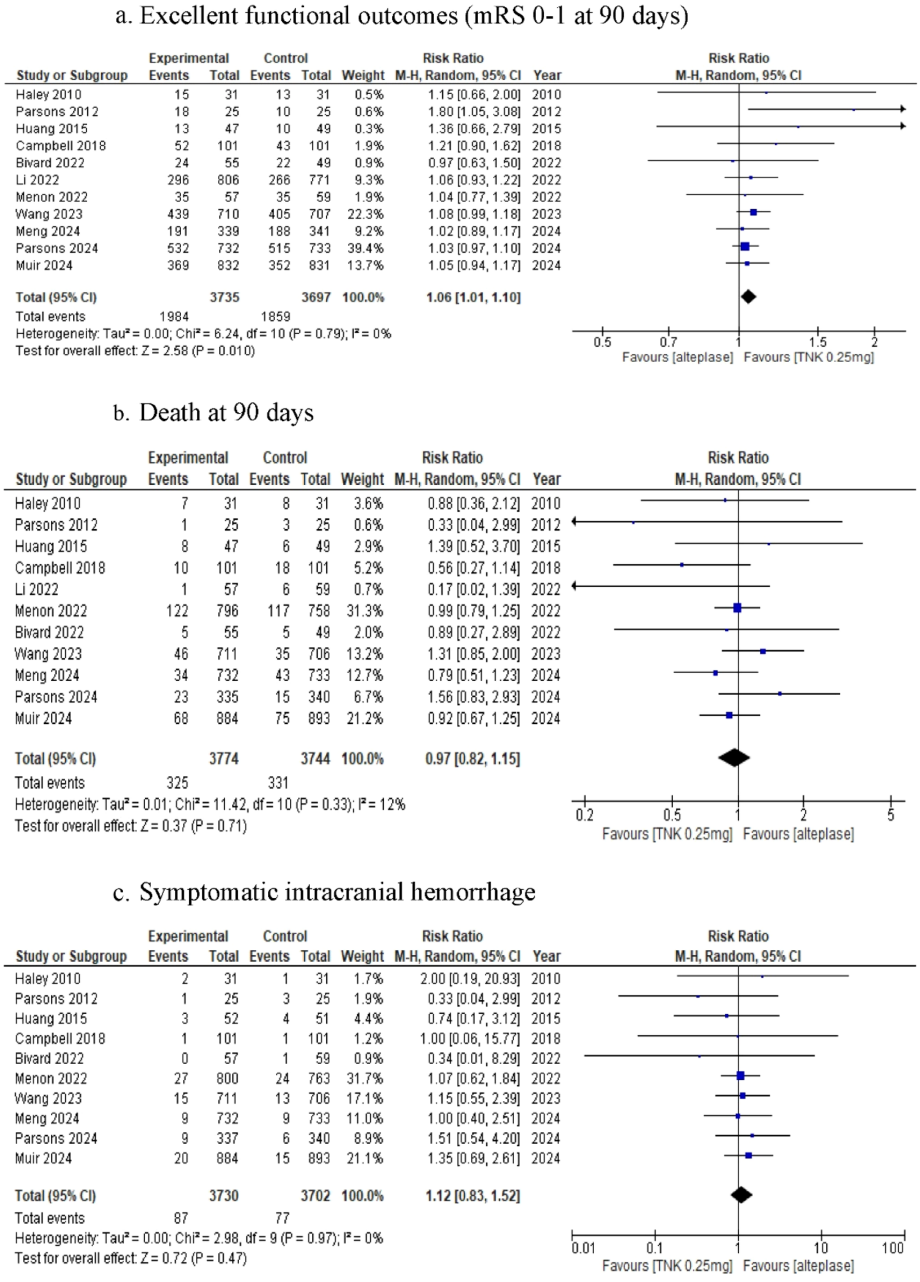
Forest plot TNK 0.25 mg/kg vs alteplase in a/Excellent functional outcome (mRS 0–1 at 90 days), b/death at 90 days and c/symptomatic intracranial hemorrhage (sICH)

For the primary endpoints, patients taking 0.25 mg/kg TNK had higher rates of excellent functional outcomes than those taking alteplase did (RR: 1.06, 95% CI (1.01–1.1), p = 0.01, I^2^ 0% and P for Cochrane Q 0.79), however, there was no difference between the two groups in achieving major neurological improvement. (RR: 1.04 95% CI (0.98–1.11), P value 0.23, I^2^ 18% and P for Cochrane Q 0.29). See Fig. 2 and Table 3.

**Table 3:** Summary of meta-analysis results

Outcome	No studies	No patients in the intervention group	No patients in the control group	Risk ratio (RR)	95%CI	P value	I^2^	P Cochran Q
TNK 0.25 mg vs alteplase								
MNI	8	2884	2890	1.04	(0.98–1.11)	0.23	18%	0.29
mRS (0–1)	11	3735	3697	1.06	(1.01–1.1)	0.01	0%	0.79
mRS (0–2)	9	3657	3617	1.03	(0.99–1.07)	0.17	35%	0.14
mRS (5–6)	4	843	838	0.85	(0.63–1.15)	0.29	0%	0.41
Death	11	3774	3774	0.97	(0.82–1.15)	0.71	12%	0.33
sICH	10	3730	3702	1.12	(0.83–1.52)	0.47	0%	.97
TNK 0.1 mg vs alteplase								
mRS (0–1)	3	116	115	0.95	(0.74–1.23)	0.7	0%	0.89
Death	3	116	115	0.67	(0.29–1.59)	0.37	21%	0.28
sICH	3	116	115	0.78	(0.18–3.45)	0.74	10%	0.33
TNK 0.4 mg vs alteplase								
MNI	3	668	686	0.93	(0.68–1.28)	0.66	72%	0.03
mRS (0–1)	3	668	686	0.84	(0.57–1.24)	0.38	75%	0.02
Death	3	668	686	1.32	(0.6–2.9)	0.49	60%	0.08
sICH	3	668	686	2.27	(0.7–7.36)	0.17	41%	0.18
Any ICH	3	668	686	1.59	(0.71–3.56)	0.26	72%	0.03
TNK 0.25 mg VS TNK 0.1 mg								
mRS (0–1)	3	113	116	1.27	(0.9–1.79)	0.17	41%	0.18
Death	3	113	116	0.67	(0.09–4.77)	0.69	69%	0.04
sICH	3	113	116	0.9	(0.13–6.20)	0.91	26%	0.26

mRS (0–1): Excellent functional outcomes

mRS (0–2): Favorable functional outcomes

mRS (5–6): Poor functional outcomes

sICH: symptomatic intracranial hemorrhage

any ICH: any intracranial hemorrhage

Subgroup analysis for major neurological improvements based on the study setting revealed no difference between the European and Chinese populations. In both groups, TNK 0.25 mg/kg was slightly better than alteplase with no statistically significant difference (RR 1.03, 95% CI (0.96–1.11), P value 0.41), and (RR 1.04, 95% CI (0.94–1.16), P value 0.45), for both European and Chinese patients. See file 1 in the supplementary materials.

Another subgroup analysis for major neurological improvement based on its definition across studies. There was no difference between TNK 0.25 mg/kg and alteplase for both definitions, either a ≥ 8 points reduction or a ≥ 4 points reduction (P values 0.35 and 0.45, respectively). See file 1 in the supplementary materials.

TNK 0.25 mg/kg had no statistically significant effect on mortality or symptomatic intracranial hemorrhage rates (sICH) (RR: 0.97, 95% CI (0.82–1.15) P value 0.71, I^2^ 12% and P for Cochrane Q 0.33), and (RR: 1.12, 95% CI (0.83–1.52) P value 0.47, I^2^ 0% and P for Cochrane Q 0.97). See Fig. 2 and Table 3.

Most studies included patients who would be eligible for mechanical thrombectomy for better interpretation of all patients with acute ischemic stroke; however, Campbell’s 2018 study was based on a comparison between 0.25 mg/kg TNK and alteplase before mechanical thrombectomy, and their results revealed that 0.25 mg/kg TNK was better than alteplase in achieving early recanalization (P value 0.04) with no statistically significant difference in achieving excellent functional outcomes, major neurological improvements, decreased death rates, or symptomatic intracranial hemorrhage (sICH) rates (P values 0.2 and 0.7,0.049 and 0.9, respectively).

TNK 0.4 mg/kg had worse safety outcomes than alteplase, as it increased death and symptomatic intracranial hemorrhage rates (RR: 1.32, 95% CI (0.6–2.9) P value 0.49, I^2^ 60% and P for Cochrane Q 0.08), (RR: 2.27, 95% CI (0.7–7.36) P value 0.17, I^2^ 41% and P for Cochrane Q 0.18). However, with small studies, no definite conclusions can be drawn.

### Sensitivity analysis

A leave-one-out test was used for all outcomes comparing 0.25 mg/kg TNK with alteplase, revealing that there is no single study that can change the significance of the outcome. (See file 1 supplementary material). However, for major neurological improvement, Parson [28] was a source of heterogeneity as after removing it, Cochran's Q p value changed from 0.03 to 0.29 and I^2^ changed from 53 to 18%. This may be because Parsons [28] included patients with stroke up to 12 h after onset. Interestingly, change in the definitions of major neurological improvements did not affect the homogeneity of the studies.

In the outcomes that compared 0.1 mg/kg TNK, no single study changed the significance of the results. On the other hand, Logallo [13] appears to be the source of heterogeneity for outcomes comparing TNK 0.4 mg/kg, and removing this study will make the study significant for alteplase; this work for major neurological improvements, excellent functional outcomes, and symptomatic intracranial hemorrhage (sICH), and for death outcomes, Kvistad [31] appeared to be the source of heterogeneity. This heterogeneity may be due to the differences in the baseline value of Kvistad [31] and Logallo [13], as Logallo [13] included patients with a lower mean age than did Kvistad [31], and 15% of patients were above 80 years of age in Logallo 2017, whereas 31% for were above 80 years of age in Kvistad [31]. Finally, 45% of patients had hypertension and 11% had hypercholesterolemia in Logallo [13], whereas 56% had hypertension and 30% had hypercholesterolemia in Kvistad [31]. Overall, no studies were excluded because a few small studies compared 0.4 mg/kg TNK with alteplase.

### Meta-regression

**Fig. 3:**
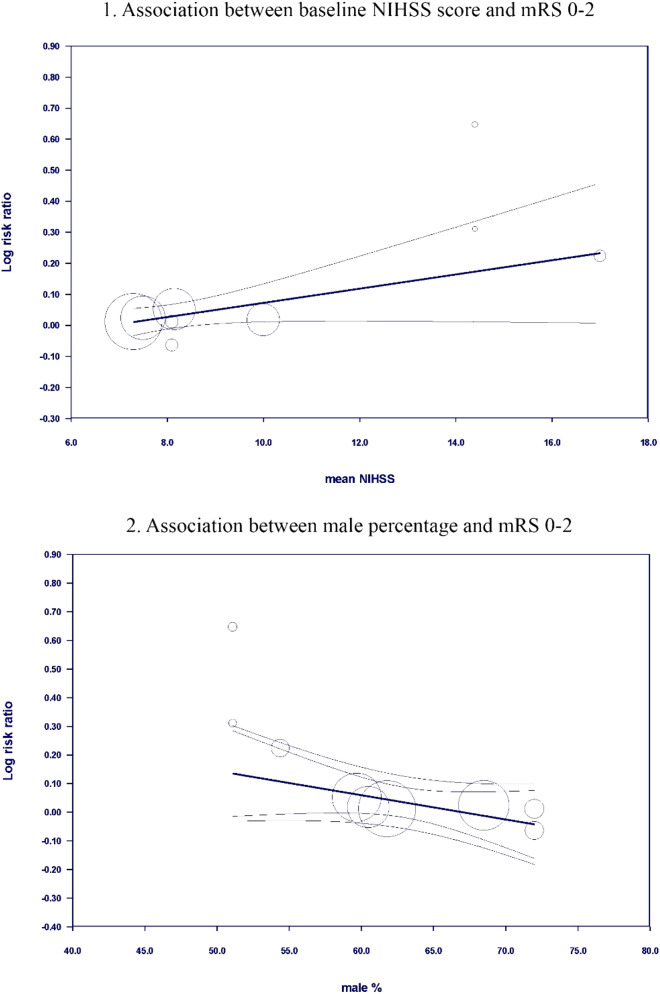
bubble plot assesses the association between 1/baseline NIHSS score and mRS0-2 and 2/male sex and mRS 0–2

We conducted a meta-regression assessing the baseline mean NIHSS score, mean age, and sex hypertension on the log risk ratio of outcomes that had 10 studies or more. We found that an increase in the baseline NIHSS score had a statistically significant negative effect on achieving favorable functional outcomes (mRS 0–2) (SE 0.1 95% CI (0.092–0.04) P value 0.025), and a male percentage increase had a negative effect on favorable functional outcomes but with not statistically significant (SE 0.0048 95% CI (− 0.018–0.0009) P value 0.08). See Fig. 3 and file 2 of the supplementary materials.

### Publication bias

Outcomes with 10 studies or more were assessed for publication bias using a funnel plot and Egger’s test, both of which were generated from the comprehensive meta-analysis version 3.7. No publication bias was observed via the funnel plot, and the results were confirmed by Egger’s test (*p* values of 0.07, 0.3, and 0.2) for mRS 0–1, death, and sICH, respectively.

## Discussion

This study is the most up-to-date in assessing the safety and efficacy of TNKs in comparison with alteplase in patients with acute ischemic stroke within 4.5 h of onset.

Our findings demonstrate the superiority of 0.25 mg/kg TNK to alteplase in achieving excellent functional outcomes at 90 days and are not inferior in achieving major neurological improvements and favorable functional outcomes, with efficacy favored by 0.25 mg/kg TNK. In addition, 0.25 mg/kg TNK did not significantly reduce death and symptomatic intracranial hemorrhage.

On the other hand, our results suggests that 0.1 mg/kg TNK had a greater safety profile than 0.25 mg/kg TNK, and the 0.4 mg/kg TNK had greater incidence of symptomatic intracranial hemorrhage than alteplase did: however, with few studies evaluating these two doses, no conclusion could be drawn.

Finally, seven of the thirteen studies had some concerns due to randomization error or deviation from the intended intervention. This puts some restrictions on the significance of the study results. Several randomized controlled trials are needed with a low risk of bias to further assess the safety and efficacy of tenecteplase (TNK) as compared to alteplase to make the results more comprehensive.

Our results are similar to those of previous systematic reviews and meta-analyses that compared different doses and different conditions of TNK with alteplase. In Wu et al. 2024, the researchers compared TNK with alteplase in patients undergoing mechanical thrombectomy, and included 10 studies, of which two were RCTs and eight were cohort studies, with 3722 patients in the alteplase group and 1,266 in the TNK group. The results revealed that TNK was superior to alteplase in terms of early recanalization with a reduced death rate and was not inferior in symptomatic intracranial hemorrhage (sICH) [33]. Another meta-analysis comparing Asian and Caucasian populations included 34 articles between RCTs and cohorts with 59,601 patients; TNK: 12,546 and Alteplase: 47,055 patients reported similar results. The early recanalization rate was high in Asian populations. A comparison of TNK doses revealed that a 0.25 mg/kg dose was associated with excellent functional outcome (mRS 0–1), whereas TNK 0.1 mg/kg was better for early recanalization, major neurological improvement, and symptomatic intracranial hemorrhage (sICH) compared to alteplase [34]. The other two meta-analyses, one focused on phase III studies and the other focused on a dose of 0.25 mg/kg, and both had similar results [35, 36]. Our study introduced more information by performing a meta-regression to determine the relationships between the baseline characteristics of the NIHSS score and sex and the study outcomes.

In a previous network meta-analysis, Srisurapanont 2024 compared different doses of TNK vs alteplase, and did not prove the superiority of TNK over alteplase in terms of the mRS score of 0–1, although they also reported no difference in other efficacy outcomes compared with our findings. They did not find statistically significant differences between the two drugs in terms of safety; however, they reported that 0.4 mg/kg TNK was associated with a greater rate of symptomatic intracranial hemorrhage (sICH). They also did not find enough data to compare different doses of TNK, but they suggested that a 0.25 mg/kg dose may be optimal. These results may be because they did not include articles published in 2024 [37].

Several studies have compared TNK with alteplase using real-world data. Warach et al., [38], focused on symptomatic intracranial hemorrhage (sICH) among 1925 patients in the TNK group and 7313 in the alteplase group and reported a reduction in the number of sICH patients in the TNK group as compared to the alteplase group [38]. Liu et al. [39] reported the same endpoint as Warach et al. [38] did in a study in China, with 1113 patients in the TNK group and 2360 patients in the alteplase group, with a median TNK dose of 0.25 mg/kg. Although they showed that TNK is safe in practice, there are concerns related to their results, as TNK group patients were significantly younger than Alteplase group patients with minor stroke [39]. Finally, Skärlunda et al. 2024 compared 888 patients receiving 0.4 mg/kg TNK with 6560 alteplase patients, and the findings were consistent with our results that 0.4 mg/kg TNK is inferior to alteplase [40].

Several trials have compared tenecteplase and alteplase as bridging therapies for mechanical thrombectomy (NCT06658197, NCT04454788, NCT05105633, NCT05199194, NCT06221371, and NCT05701956), and Qiu et al. [41]. These trials will enhance the current knowledge for a better understanding of the safety and efficacy of TNK.

Other trials have assessed the safety and efficacy of TNKs as intra-arterial injections for large vessel occlusion (NCT05657470, NCT05657444, and NCT05657457 [42]). Recently, two trials, Huang 2025 and Hu 2025, have been published discussing intra-arterial TNKs in comparison with controls. Both included 747 patients, of whom 373 patients were treated by intra-arterial TNKs and the remaining patients were in the control group. They reported a slightly higher rate of excellent functional outcomes, with no statistically significant results, however, Huang 2025 reported a statistically significant increase in symptomatic intracranial hemorrhage (sICH) in the TNK group [43, 44]. Further studies should be conducted to better understand its effect.

In addition, several studies have assessed the efficacy of TNK in the extended window; for example, a recent systematic review and meta-analysis of three small sample size RCTs compared TNK versus standard medical treatment for patients with stroke after 4.5 h of onset which showed statistically significant excellent functional outcomes (P value 0.04), with some concern of safety as the results tended to be better in the control groups; however, owing to the small sample size, no definite conclusions could be reached. The recent RCTs in this area have been conducted [45–47]. Their results are variable. Xiong 2024 reported a significantly better performance of TNK than the best medical management and excellent and favorable functional outcomes, with a better safety profile, with no statistical significance [45]. However, Coutts 2024 reported that the best medical management was better than the TNK group in terms of safety and efficacy profiles, with fewer adverse effects in the best medical management group [47]. This may be because Coutts 2024 included patients with minor ischemic stroke. Further RCTs are needed to further evaluate TNK usage in this field.

## Strengths and limitations

The study has several strengths as it is the most up-to-date on this important topic. We included randomized control trials in different ethnicities to strengthen our evidence. Our study found that TNK is superior to alteplase in achieving excellent functional outcomes and is not inferior in terms of safety profile. Therefore, ischemic stroke management guidelines further recommend 0.25 mg/kg TNK as an alternative superior choice to alteplase (tPA). This will make managing acute ischemic stroke more flexible if alteplase is not available.

On the other hand, some limitations are present; we include other doses of TNK, which are TNK 0.1 mg/kg and 0.4 mg/kg does. These doses lack sufficient data to interpret their results, so we cannot make a final conclusion about their results unless other randomized controlled trials are conducted. In addition, seven RCTs had some concerns related to quality assessment, limiting the reliability of their results. Another limitation is the wide range of confidence intervals for symptomatic intracranial hemorrhage (sICH) when TNK 0.25 mg/kg is compared with alteplase, which makes it difficult to make a definitive conclusion. Finally, a previous systematic review has been published in this scope with similar results.

## Conclusion

We present the most up-to-date study to compare different doses of TNK versus alteplase in patients with acute ischemic stroke and demonstrate that 0.25 mg/kg TNK provides the best overall outcomes. Compared with alteplase, 0.25 mg/kg TNK is superior in terms of excellent functional outcomes and is not inferior in terms of major neurological improvements and safety outcomes. This conclusion is based on the significance of the outcome P values and their corresponding confidence interval. The current knowledge supports that 0.25 mg/kg TNK is the best dose option in achieving efficacy and safety for the treatment of acute ischemic stroke to date. The available data suggest that TNK 0.1 and 0.4 doses are not good options; however, several RCTs are needed to confirm this. Currently, 0.25 mg/kg TNK can be administered safely for patients with acute ischemic stroke within 4.5 h of onset, with better outcomes than alteplase.

## Supplementary Information

Additional file 1

Additional file 2

## Data Availability

No datasets were generated or analysed during the current study.

## Abbreviations

TNK: Tenecteplase
tPA: Alteplase
AIS: Acute ischemic stroke
NIHSS: National institutes of health stroke scale
mRS: Modified Rankin score
sICH: Systemic intracranial hemorrhage
ECASS: European cooperative acute stroke study
ESO: European stroke organization
STEMI: ST-segment elevation myocardial infarction
PCI: Percutaneous coronary intervention
