# Supplementary material for: Tenecteplase versus alteplase in patients with acute ischemic stroke: an updated systematic review and meta-analysis
Source: Eur J Med Res. 2025 Aug 8;30:726. doi: 10.1186/s40001-025-02983-9 (PMC12333306; doi:10.1186/s40001-025-02983-9)
Supplement: Supplementary file 1 — Additional file 1 [file 40001_2025_2983_MOESM1_ESM.docx]

Tenecteplase versus alteplase in patients with acute ischemic stroke: an updated systematic review and meta-analysis

Abdelmonam M Hagag^1^ [
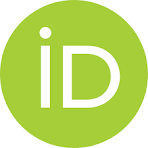
](https://orcid.org/0009-0008-1392-3505), Muhammed E. Kormod^2^[
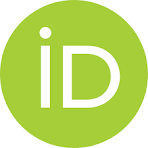
](https://orcid.org/0009-0004-9036-3338), Mahmoud Elmwafy Ads^3^, Mennatullah A. El-Refaay^4^[
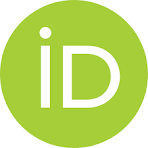
](https://orcid.org/0009-0006-2155-5082),Omnia M. Abozaid^5^[
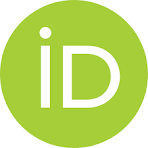
](https://orcid.org/0009-0003-9841-0146)^,^ Omar A. Ghanem^1^[
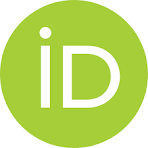
](https://orcid.org/0009-0009-1966-3705)^,^ Mazen Yasser^1^[
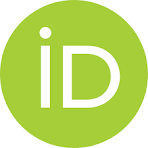
](https://orcid.org/0009-0005-2090-2073)^,^ Karim M. Abdelmoaty^1^, Alaa Mahmoud Khedr^1^[
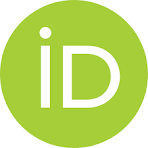
](https://orcid.org/0009-0003-6213-4936)^,^ Gregory W Albers^6^ [
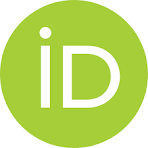
](https://orcid.org/0000-0003-0263-4632)

1. Faculty of Medicine, Zagazig University, Zagazig, Egypt
2. Faculty of Medicine, Al-Azhar University, Cairo, Egypt
3. Faculty of Medicine, Mansoura University, Mansoura, Egypt
4. Faculty of Medicine, October 6 University, Cairo, Egypt
5. Faculty of Medicine, Minia University, Minia, Egypt
6. Department of Neurology, Stanford University, Palo Alto, CA, United States

**Corresponding author:** Abdelmonam M Hagag

**Affiliation**: Faculty of Medicine, Zagazig University, Zagazig, Egypt

Email: [abdelmonamhagag@gmail.com](mailto:abdelmonamhagag@gmail.com)

Translational stroke research

| object | Page number |
| --- | --- |
| [**Title, authors’ information**](#Bookmark1) | 1 |
| [**Table S1**](#Bookmark2) | 2 |
| [**Quality assessment results**](#Bookmark4) | 5 |
| [**TNK 0.25 mg vs alteplase**](#Bookmark10) | 5 |
| [**subgroup analysis for TNK 0.25 mg/kg vs alteplase**](#Bookmark6) | 8 |
| [**Leave-one-out test**](#Bookmark11) | 8 |
| [**TNK vs alteplase**](#Bookmark9) | 11 |
| [**TNK 0.25 mg VS TNK 0.1 mg**](#Bookmark8) | 16 |
| [**Publication bias**](#Bookmark7) | 17 |

| **Table S1:** **Search Strategy** | | | |  |  |
| --- | --- | --- | --- | --- | --- |
| Search strategy used for all the databases | | (Tenecteplase OR Metalyse OR TNKase OR TNK tPA OR Elaxim) AND (Alteplase OR Actilyse OR Activase OR Cathflo OR Cathflo Activase OR Tissue plasminogen activator OR TTPA OR Lysatec rt-PA OR Lysatec rtPA OR Tissue Activator D 44 OR Tissue-Type Plasminogen Activator OR Tisokinase OR T Plasminogen Activator OR Plasminogen Activator, Tissue-Type OR TPA) AND (Ischemic stroke OR Ischemic Strokes OR Ischaemic Stroke OR Acute Ischemic Stroke OR Cryptogenic Ischemic Stroke OR Embolism Stroke, Cryptogenic OR Cryptogenic Stroke OR Wake-up Stroke OR Brain Ischemias OR Cerebral Ischemia OR TIA OR transient ischemic attack OR Cerebral Infarction OR Middle Cerebral Artery Infarction OR Middle Cerebral Artery stoke OR Thrombectomy) | |  |  |
| **Table S1: Summary of the results** | | | | | |
| **Study ID** | | **Inclusion criteria** | | **Results** | |
| **Haley 2010** | | - patients were aged ≥18 years - serious neurological deficits believed to be based on acute focal cerebral ischemia - suitable for treatment with intravenous rtPA within 3 hours of stroke onset using contemporary guidelines | | The 0.4-mg/kg dose was discarded as inferior, the selection procedure was still unable to distinguish between 0.1 mg/kg and 0.25 mg/kg as a propitious dose.  Symptomatic intracranial hemorrhage rates were highest in the discarded 0.4-mg/kg tenecteplase group and lowest (0 of 31) in the 0.1-mg/kg tenecteplase group.  The study did not find such strong evidence to show meaningful differences between the tenecteplase groups and the rtPA group in terms of 3-month outcomes. | |
| **Parsons 2012** | | - Patients with first-ever hemispheric ischemic stroke - aged 18 or older - NIHSS score greater than 4 - premorbid Rankin scale score of 2 or less. - Specific CT imaging criteria: presence of a perfusion lesion at least 20% greater than the infarct core and associated vessel occlusion. | | Together, the two tenecteplase groups had greater reperfusion (P=0.004) and clinical improvement (P<0.001) at 24 hours than the alteplase group.  Intracranial bleeding or other serious adverse events were with no significant differences between groups.  The higher dose of tenecteplase (0.25 mg/kg) was superior to the lower dose (0.1 mg/kg) and to alteplase for all efficacy outcomes | |
| **Huang 2015** | | - Adults with clinically diagnosed supratentorial ischemic stroke - measurable deficit on NIHSS - within 4.5 hours of onset, living independently pre-stroke. | | Neurological and radiological outcomes did not differ between two groups.  A larger core volume and higher artery occlusion rates were observed in the tenecteplase group, but these differences were not statistically significant.  71 patients had satisfactory imaging for the primary outcome, with no significant difference in penumbra salvaged or secondary endpoints between treatment groups. In a post-hoc analysis adjusting for core volume and occlusion site, no significant impact was observed on primary outcomes or recanalization rates.  For safety, intracerebral hemorrhage occurred in 15% of the tenecteplase group and 27% of the alteplase group (p=0.09). Parenchymal hemorrhage occurred in 2% of tenecteplase patients versus 10% in the alteplase group. Symptomatic intracerebral hemorrhage rates were similar between groups. Serious adverse events occurred in 42% of the tenecteplase group and 31% of the alteplase group. | |
| **Logallo 2017** | | - Adults with clinically suspected acute ischemic stroke - Admitted within 4.5 hours of symptom onset or within 4.5 hours of awakening with symptoms - Eligible for intravenous thrombolysis or bridging therapy before thrombectomy | | Tenecteplase was not superior to alteplase and had a similar safety profile.  Intracranial hemorrhage occurred in 9% of patients in both groups, and symptomatic intracranial hemorrhage rates were also similar (3% for tenecteplase and 2% for alteplase; p=0.70). Mortality rates were 5% in both groups, and no differences were observed in major neurological improvement, secondary outcomes, or serious adverse events.  The per-protocol analysis of 773 patients also showed no significant differences, with 64% achieving excellent functional outcomes in both groups. Serious adverse events occurred in 26% of patients in both groups, with intracranial hemorrhage being the most frequent. There were similar numbers of hospital readmissions up to day 90. | |
| **Campbell 2018** | | - Could undergo intravenous thrombolysis within 4.5 hours after the onset of ischemic stroke - Had a large-vessel occlusion in the internal carotid artery, middle cerebral artery, or basilar artery. - Were eligible for endovascular thrombectomy | | The primary outcome was substantial reperfusion, defined as more than 50% reperfusion of the ischemic territory at the first angiographic assessment. Difference was statistically significant, with tenecteplase showing a higher rate of reperfusion (P = 0.03 for superiority).  Symptomatic intracerebral hemorrhage (sICH) occurred in 1% of patients in both the tenecteplase and alteplase groups.(2ry outcome)  The proportion of patients with a favorable functional outcome (modified Rankin scale score of 0-2) at 90 days was:  64% for tenecteplase group, 55% for alteplase group  Although the tenecteplase group showed a numerically better outcome, this difference was not statistically significant (P = 0.06). | |
| **Menon 2022** | | - Patients aged 18 years or older - a diagnosis of acute ischemic stroke, causing disabling neurological deficit - presenting within 4.5 hours of symptom onset - eligible for thrombolysis | | AcT trial provides robust empirical evidence that tenecteplase is comparable to alteplase in patients presenting with acute ischemic stroke, with a similar function, quality of life, and safety outcomes. Given the ease of administration of tenecteplase compared with alteplase, these results provide a compelling rationale to support switching the standard-of-care intravenous thrombolytic agent for acute ischemic stroke from alteplase to tenecteplase at a dose of 0·25 mg/kg.  Tenecteplase achieved a modified Rankin Scale (mRS) score of 0-1 at 90-120 days in 36.9% of patients, compared to 34.8% for alteplase. This met the non-inferiority margin, showing that tenecteplase is non-inferior to alteplase.(primary outcome)  Rates of mRS scores of 0-2 were similar between tenecteplase (56.4%) and alteplase (55.6%). The need for endovascular thrombectomy and other functional outcomes showed no significant differences between the groups.(2ry outcome)  Symptomatic intracerebral hemorrhage within 24 hours occurred in 3.4% of tenecteplase patients and 3.2% of alteplase patients. The 90-day mortality rate was almost identical between the two groups (15.3% for tenecteplase vs. 15.4% for alteplase). | |
| **Li 2022** | | - Patients aged 18 years or older - diagnosed of AIS with measurable deficits on the NIHSS 4–25 (both included) - hemorrhage ruled out by non-contrast computer tomography (NCCT) scan - admitted within 3 hours of symptom onset which refers to ‘last known to be well - living independently (pre-stroke modified Rankin Scale (mRS)≤2 or without history of stroke) - had no contraindications to intravenous thrombolysis | | Results showed that intravenous rhTNK-tPA, given within 3 hours of symptom onset, is a well-tolerated option in patients with AIS in China. A dose of 0.25 mg/kg may be suggested for future efficacy studies in Caucasians patients with AIS based on the results of contemporary clinical studies. The efficacy dose of rhTNK-tPA in East Asians needs to be investigated with further investigation.  No significant difference in primary efficacy was observed between the tenecteplase groups and the alteplase group.(1ry outcome)  There were no statistically significant differences between any of the groups for mRS outcomes.(2ry outcome)  for the safety outcome no significant differences in sICH or death were observed across groups. | |
| **Kvistad 2022** | | - clinically suspected acute ischaemic stroke in patients aged 18 years or older - living independently before their stroke - admitted within 4·5 h of stroke onset with an NIHSS score of 6 or more - eligible for thrombolytic treatment according to Norwegian guidelines - Patients with signs or symptoms on awakening or an unknown onset of stroke signs or symptoms were included if an MRI showed a mismatch between diffusion-weighted imaging and fluid-attenuated inversion recovery - Patients undergoing a thrombectomy were included if they received thrombolysis as a bridging  therapy | | The study consequently could not show that 0·4 mg/kg tenecteplase is non-inferior to alteplase in moderate and severe ischaemic stroke.It resulted in worse functional outcomes and a higher risk of intracranial hemorrhage and mortality compared to standard-dose alteplase in patients with moderate to severe ischemic stroke. Future stroke trials should assess a lower dose of tenecteplase versus alteplase in patients with moderate or severe stroke.  A favorable functional outcome (modified Rankin Scale score of 0-1) at 3 months was observed in 31 out of 96 patients (32%) receiving tenecteplase, compared to 52 out of 101 patients (51%) receiving alteplase. The odds ratio was 0.45 (95% CI 0.25–0.80, p=0.0064), indicating a statistically significant lower efficacy for tenecteplase.(1ry outcome)  Major neurological improvement at 24 hours was less frequent in the tenecteplase group (58%) compared to the alteplase group (74%, OR 0.48, p=0.018). The analysis also showed that patients treated with tenecteplase had poorer functional outcomes overall, with a higher proportion having a modified Rankin Scale score of 5 or 6 (18% vs. 6%).(2ry outcome)  regarding the safety outcomes the incidence of any intracranial hemorrhage was significantly higher in the tenecteplase group (21% vs. 7% for alteplase, OR 3.68, 95% CI 1.49–9.11, p=0.0031). Symptomatic intracranial hemorrhage occurred in 6% of tenecteplase patients and 1% of alteplase patients (p=0.061). Mortality at 3 months was also higher in the tenecteplase group (16% vs. 5%, OR 3.56, 95% CI 1.24–10.21, p=0.013). | |
| **Bivard 2022** | | - were aged 18 years or older - patients with ischemic stroke within 4·5 h of onset - eligible for intravenous thrombolysis - independent mobility before stroke, and a pre-stroke mRS score of 3 or less | | The study administered a 0.25 mg/kg intravenous bolus of Tenecteplase compared to standard-of-care alteplase (0.9 mg/kg) and found that Tenecteplase on the MSU resulted in superior reperfusion and presented no safety concerns. Thus, the trial provides evidence to support the use of Tenecteplase and MSUs as an optimal model of stroke care. | |
| **Wang 2023** | | - aged at least 18 years - could receive intravenous thrombolytics within 4·5 h of their ischemic stroke - had a mRS score of no more than 1 before enrolment - had a disabling ischemic stroke with a NIHSS score of 5–25 | | In the TRACE-2 trial, Tenecteplase was administered at a dose 0.25 mg/kg (maximum 25 mg), while alteplase was given at 0.9 mg/kg (maximum 90 mg/kg) for acute ischemic stroke treatment. Results indicated that Tenecteplase was non-inferior to alteplase regarding functional outcomes and safety profiles. Recommendations support using Tenecteplase as a viable alternative to alteplase in clinical practice for eligible patients. | |
| **Parsons 2024** | | - aged 18 years or older - could receive an intravenous thrombolytic within 4·5 h after the onset of symptoms - had confirmed the presence of an acute ischemic stroke. - CT-perfusion target mismatch ratio greater than 1.8, Absolute mismatch difference <15 ml. Ischemic core lesion volume >70 ml. The volume of severely hypo-perfused tissue (delay time >8 seconds) >100 mL | | the TASTE trial compared Tenecteplase and alteplase for thrombolysis in patients with ischemic stroke within 4.5 hours of onset. The dose of alteplase used for comparison was 0.90 mg/kg body weight, administered as a bolus and infusion. The study indicated that Tenecteplase was non-inferior to alteplase by per-protocol analysis, and superior for excellent functional outcome at 90 days. These findings support the implementation of intravenous Tenecteplase 0.25mg/kg as the new standard of care thrombolytic agent, potentially influencing treatment recommendations for patients with disabling ischemic stroke within 4.5 hours of onset. | |
| **Meng 2024** | | - Chinese adults (aged ≥18 years) - had an AIS with NIHSS score of 1 to 25 - measurable neurologic deficit, had been symptomatic for at least 30 minutes without significant improvement - were able to receive thrombolytic therapy within 4.5 hours of symptom onset - with an NIHSS score of >4 and have a measurable deficit in motor function score for the arms or legs of at least 1 - whom endovascular thrombectomy was planned were eligible | | Tenecteplase was non-inferior to alteplase concerning excellent functional outcomes (mRS score of 0 or 1) at 90 days in patients with AIS within 4. 5 hours of symptom onset. Findings from this study provide evidence to support the use of tenecteplase as a suitable alternative to alteplase in these patients | |
| **Muir 2024** | | - Previously independent adults with an estimated modified Rankin Scale [mRS] score of 0–2 before stroke - Age ≥18 years   with acute ischemic stroke, eligible for intravenous thrombolysis less than 4·5 h from last known well | | Tenecteplase was non-inferior to alteplase for mRS score distribution at 90 days but was not superior. 68 (8%) patients in the tenecteplase group compared with 75 (8%) patients in the alteplase group died, symptomatic intracerebral hemorrhage (defined by SITS-MOST criteria) occurred in 20 (2%) versus 15 (2%) patients, parenchymal hematoma type 2 occurred in 37 (4%) versus 26 (3%) patients, post-treatment  intracranial bleed occurred in 94 (11%) versus 78 (9%) patients, significant extracranial hemorrhage occurred in 13 (1%)  versus six (1%) patients, respectively, and angioedema occurred in six (1%) participants in both groups. | |
| NIHSS: National Institutes of Health Stroke Scale  mRS: modified Rankin score  AIS: acute ischemic stroke | | | | | |

**Quality assessment results**


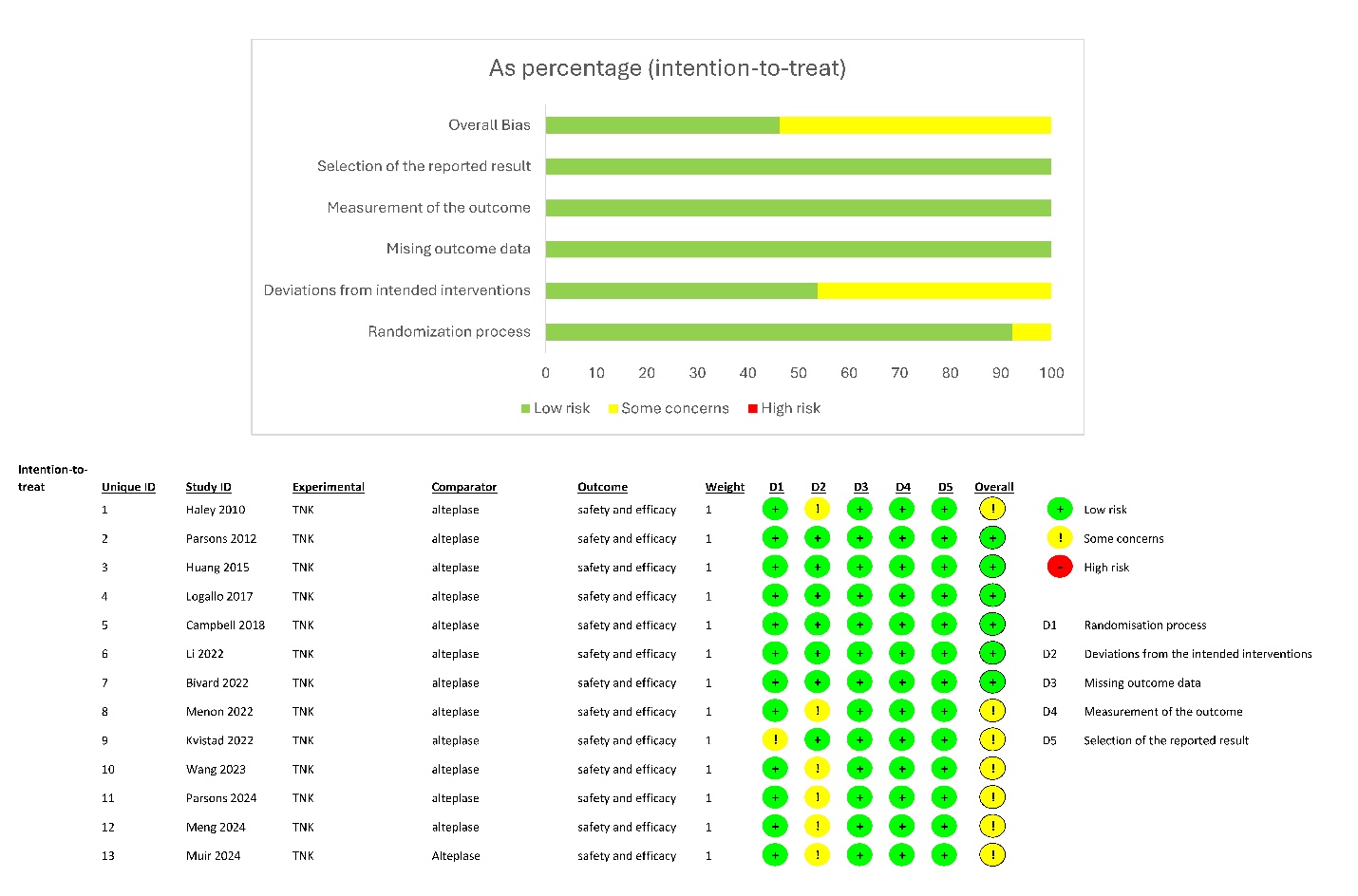


**Meta-analysis forest plots**

1. TNK 0.25 mg vs alteplase
2. MNI


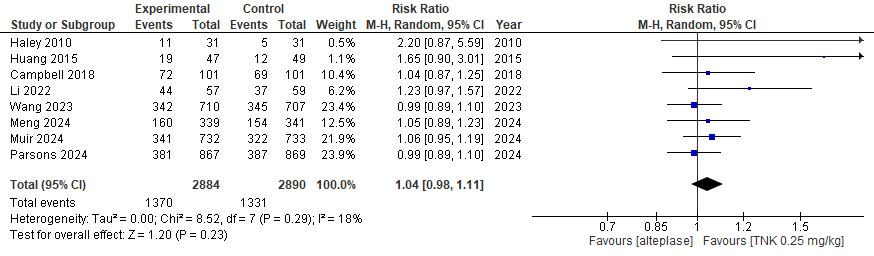


1. Excellent functional outcomes (mRS 0-1 at 90 days)


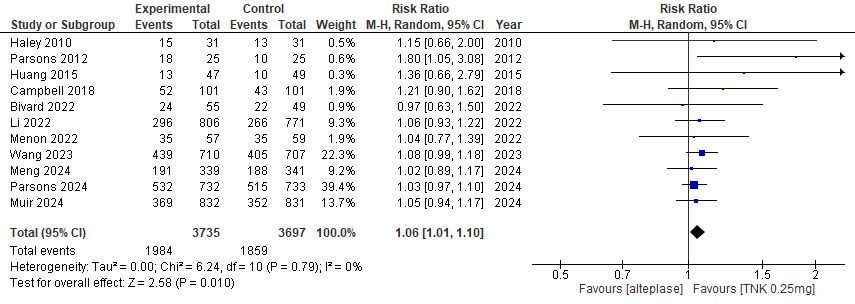


1. Favorable functional outcomes (mRS 0-2 at 90 days)


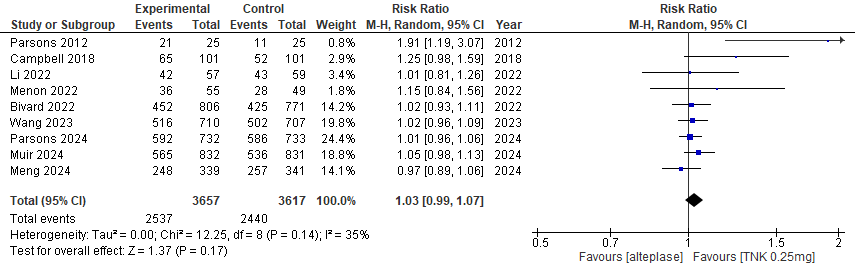


1. Poor functional outcomes (mRS 5-6 at 90 days)


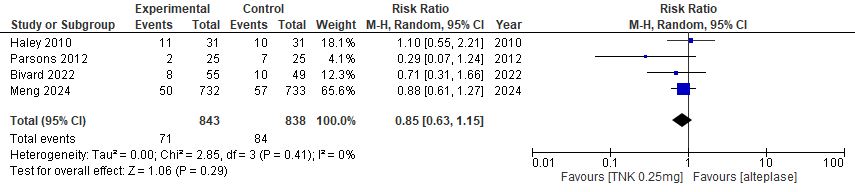


1. Death at 90 days


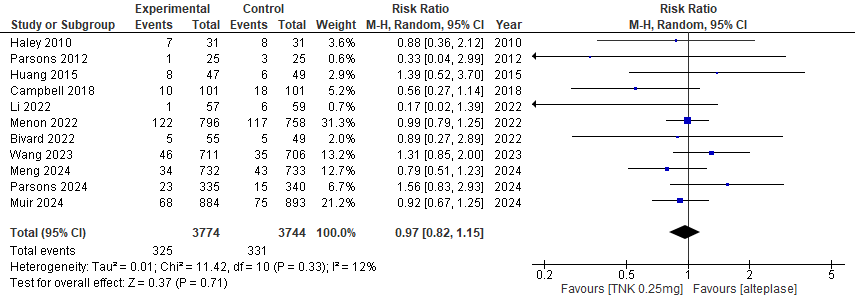


1. Symptomatic intracranial hemorrhage


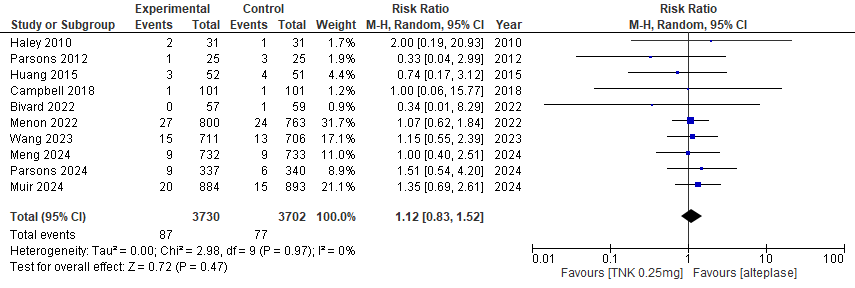


1. Any intracranial hemorrhage


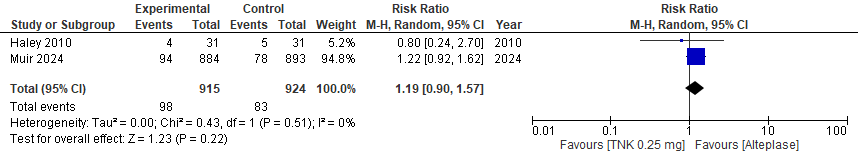


1. Subgroup analysis for TNK 0.25 mg/kg vs alteplase
2. Major neurological improvement (According to the study setting)


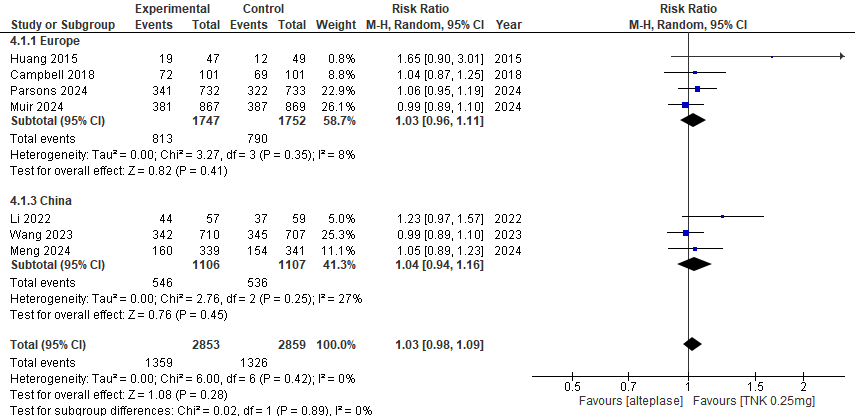


1. Subgroup analysis according to MNI definitions


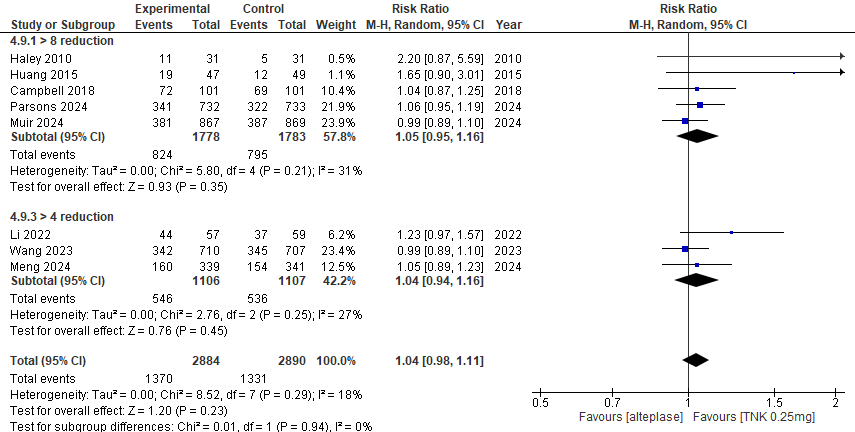


1. Leave-on-out test
2. Major neurological improvement

1. Excellent functional outcome (mRS 0-1 at 90 days)

1. Favorable functional outcome (mRS 0-2 at 90 days)

1. Poor functional outcome (mRS 5-6 at 90 days)

1. Death at 90 days

1. Symptomatic intracranial hemorrhage

TNK 0.1 mg/kg VS alteplase

1. Excellent functional outcomes (mRS 0-1 at 90 days)


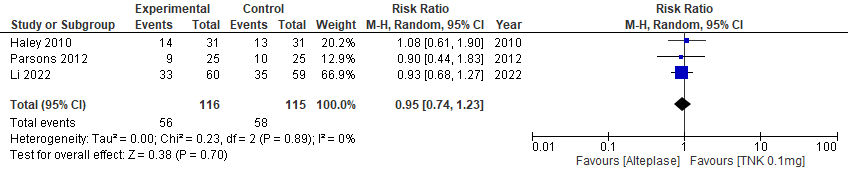


1. Death at 90 days


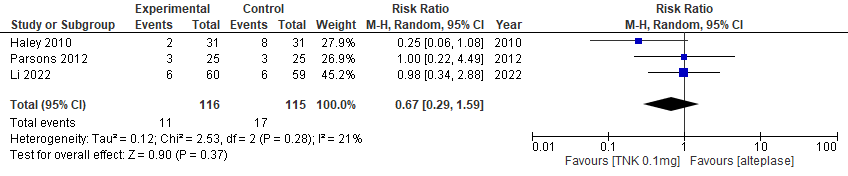


1. Symptomatic intracranial hemorrhage (sICH)


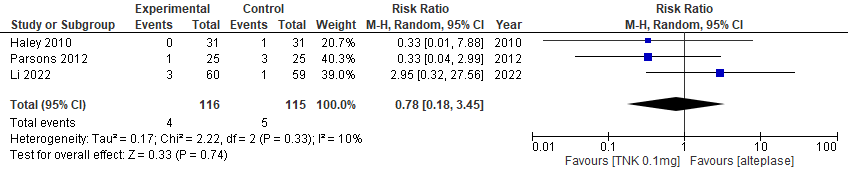


TNK 0.4 mg/kg vs alteplase

1. MNI


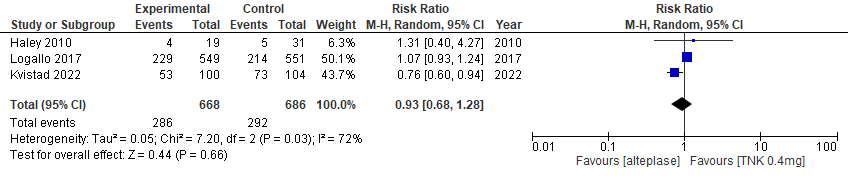


1. Excellent functional outcomes (mRS 0-1 at 90 days)


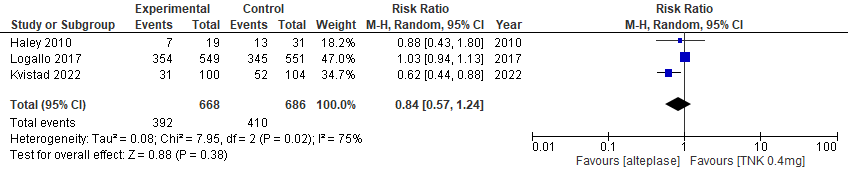


1. Death at 90 days


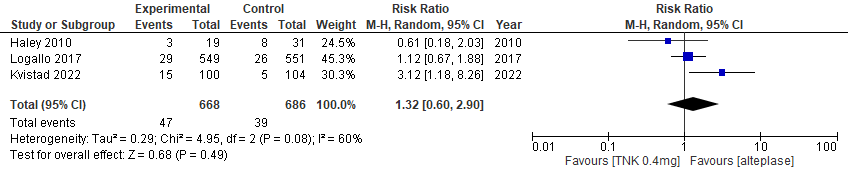


1. Symptomatic intracranial hemorrhage (sICH)


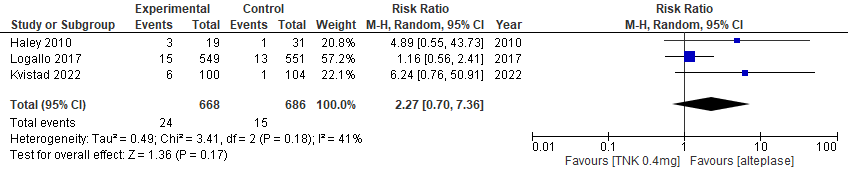


1. Any intracranial hemorrhage


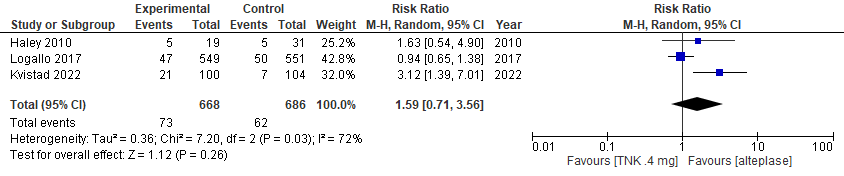


1. TNK 0.25 mg VS TNK 0.1 mg
2. Excellent functional outcomes (mRS 0-1 at 90 days)


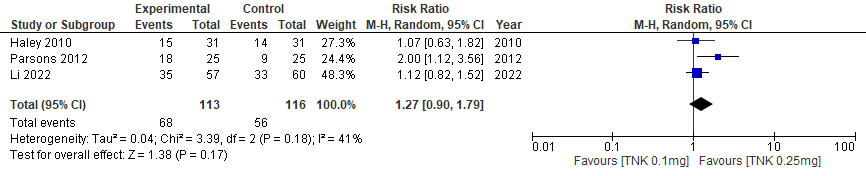


1. Death at 90 days


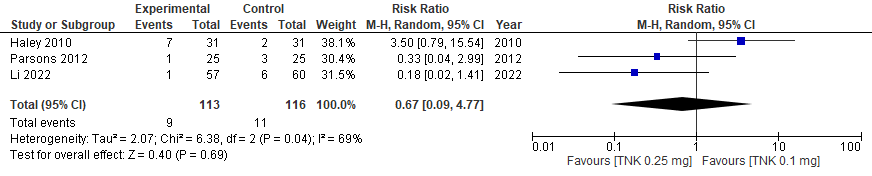


1. Symptomatic intracranial hemorrhage


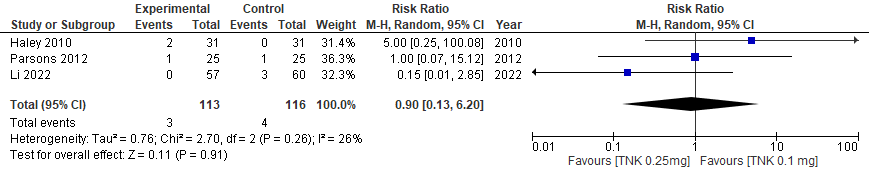


**Publication bias**

**TNK 0.25 mg/kg VS-alteplase**

- **Excellent functional outcomes (mRS 0-1 at 90 days)**

- **Death at 90 days**

- **Symptomatic intracranial hemorrhage**
