# Supplementary material for: Tenecteplase versus alteplase in patients with acute ischemic stroke: an updated systematic review and meta-analysis
Source: Eur J Med Res. 2025 Aug 8;30:726. doi: 10.1186/s40001-025-02983-9 (PMC12333306; doi:10.1186/s40001-025-02983-9)
Supplement: Supplementary file 2 — Additional file 2 [file 40001_2025_2983_MOESM2_ESM.docx]

**Meta-regression**

| Outcome | Standard error | 95%confidence interval (CI) | P value |
| --- | --- | --- | --- |
| Mean age | | | |
| mRS 0-1 | 0.001 | (-0.0008–0.0032) | 0.25 |
| mRS 0-2 | 0.0083 | (-0.01–0.012) | 0.75 |
| MNI | 0.002 | (-0.0012–0.006) | 0.2 |
| Death | 0.005 | (-0.02–0.003) | 0.2 |
| sICH | 0.006 | (-0.01–0.01) | 0.7 |
| Mean NIHSS score | | | |
| mRS 0-1 | 0.001 | (-0.0031–0.0008) | 0.2 |
| mRS 0-2 | 0.01 | (0.092–0.04) | 0.025* |
| MNI | 0.002 | (-0.006–0.003) | 0.2 |
| Death | 0.006 | (-0.003–0.02) | 0.2 |
| sICH | 0.007 | (-0.01–0.01) | 0.8 |
| Male percentage | | | |
| mRS 0-1 | 0.0046 | (-0.0057–0.023) | 0.48 |
| mRS 0-2 | 0.0048 | (-0.018–0.0009) | 0.08 |
| MNI | 0.007 | (-0.01–0.02) | 0.8 |
| Death | 0.02 | (-0.03–0.05) | 0.7 |
| sICH | 0.02 | (-0.04–0.04) | 0.96 |
| Hypertension percentage | | | |
| mRS 0-1 | 0.0027 | (-0.0035–0.0072) | 0.5 |
| MNI | 0.06 | (-0.01–0.012) | 0.85 |
| Death | 0.01 | (-0.04–0.02) | 0.4 |
| sICH | 0.02 | (-0.02–0.03) | 0.7 |
| mRS 0-1: Excellent functional outcome  mRS 0-2: Favorable functional outcome  MNI: Major neurological improvement  sICH: Symptomatic intracranial hemorrhage | | | |

**Mean Age**

mRS 0-1 (SE 0.001 95%CI (-0.0008–0.0032) P value 0.25)

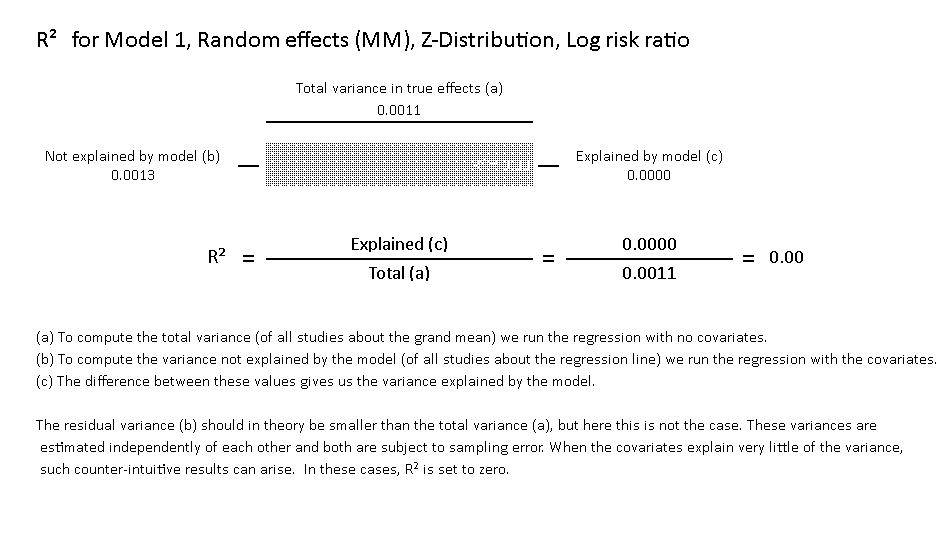


mRS 0-2 (SE 0.0083 95%CI (-0.01–0.012) P value 0.75)

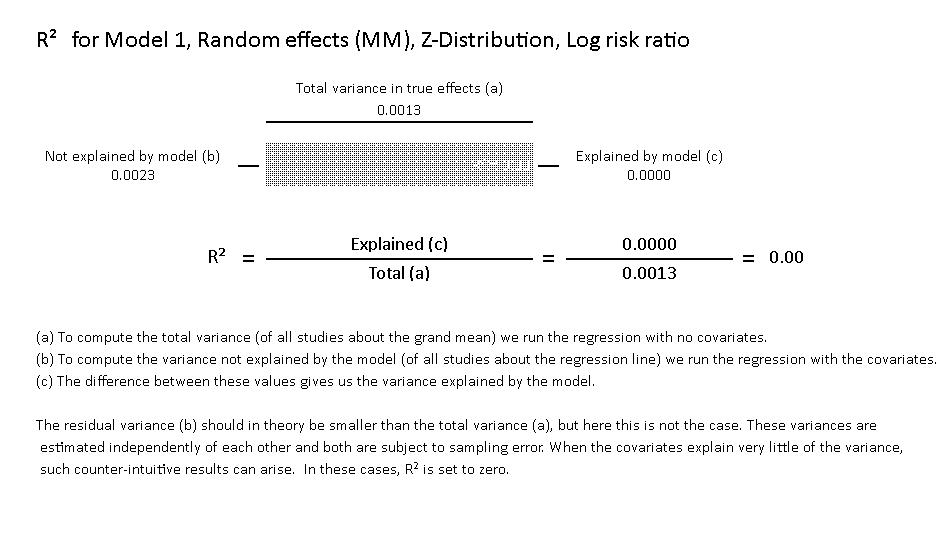


Major neurological improvement MNI (SE 0.002 95%CI (-0.0012–0.006) P value 0.2)

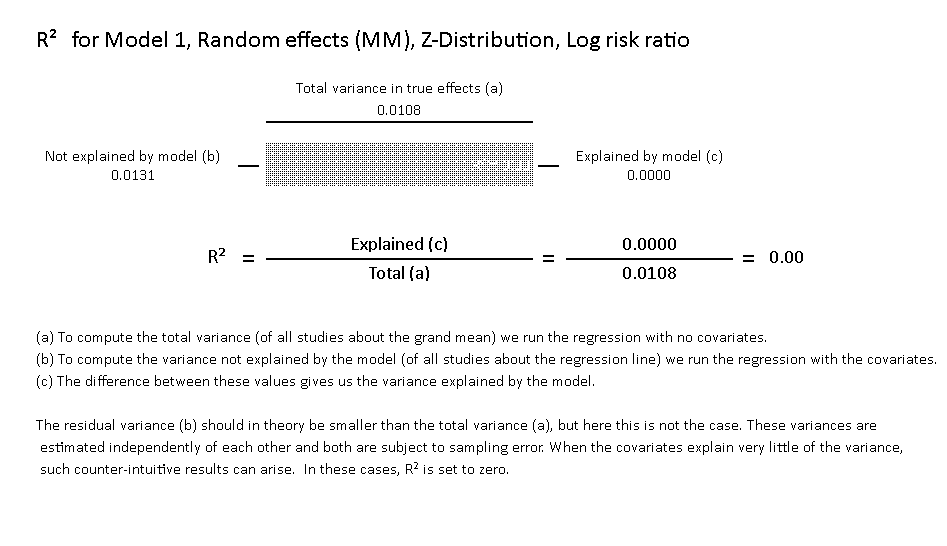


Death (SE 0.005 95%CI (-0.02–0.003) P value 0.2)

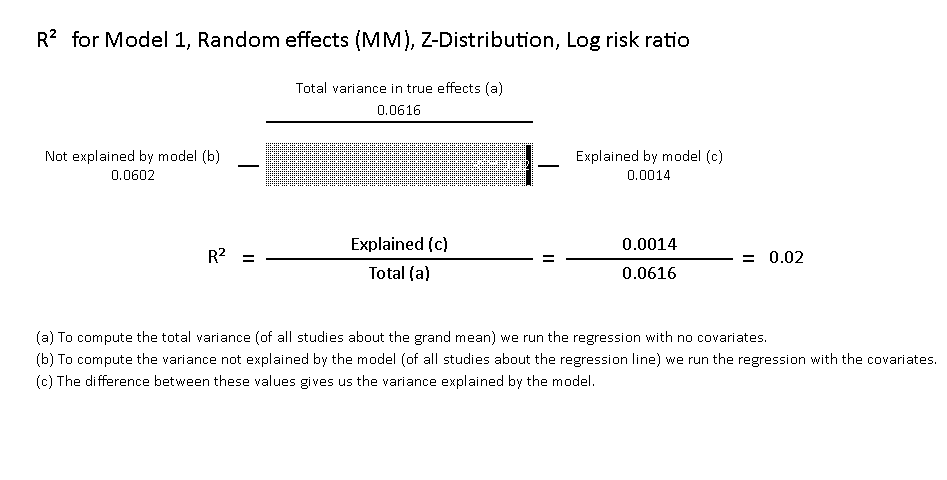


Symptomatic intracranial hemorrhage sICH (SE 0.006 95%CI (-0.01–0.01) P value 0.7)

**Mean NIHSS score**

mRS 0-1 (SE 0.001 95%CI (-0.0031–0.0008) P value 0.2)

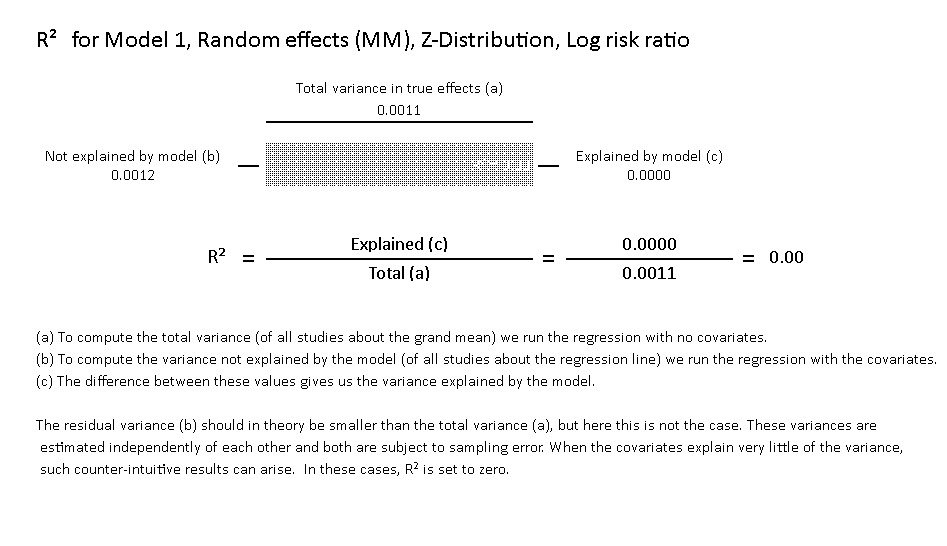


mRS 0-2 (SE 0.01 95%CI (0.092–0.04) P value 0.025)

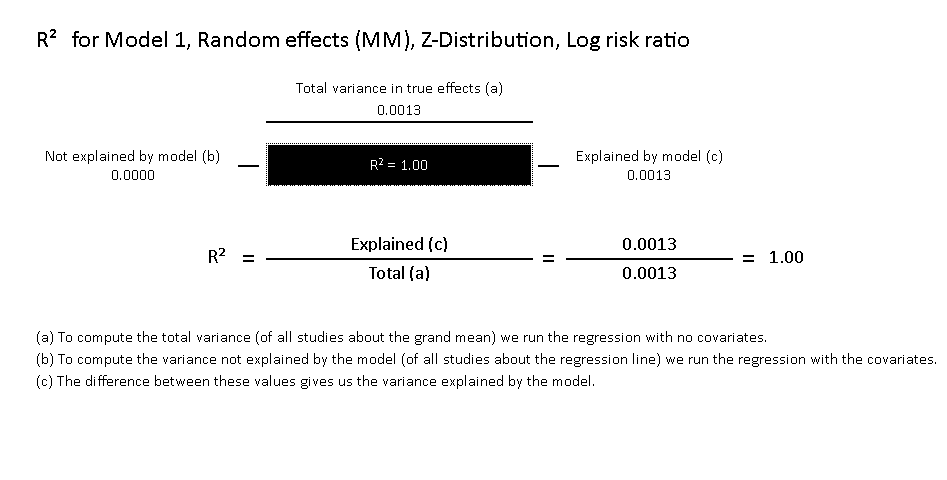


**Major neurological improvement MNI** (SE 0.002 95% CI (-0.006–0.003) P value 0.2)

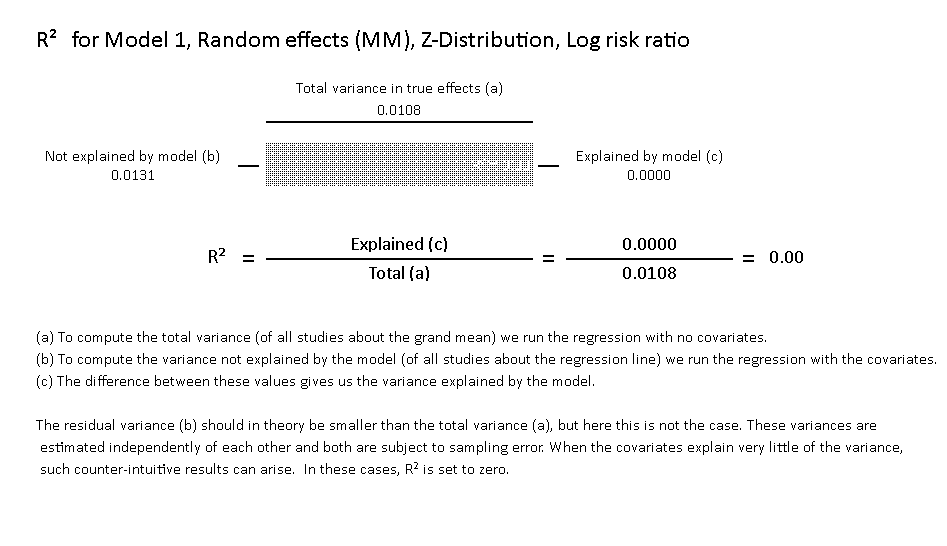


Death (SE 0.006 95% CI (-0.003–0.02) P value 0.2)

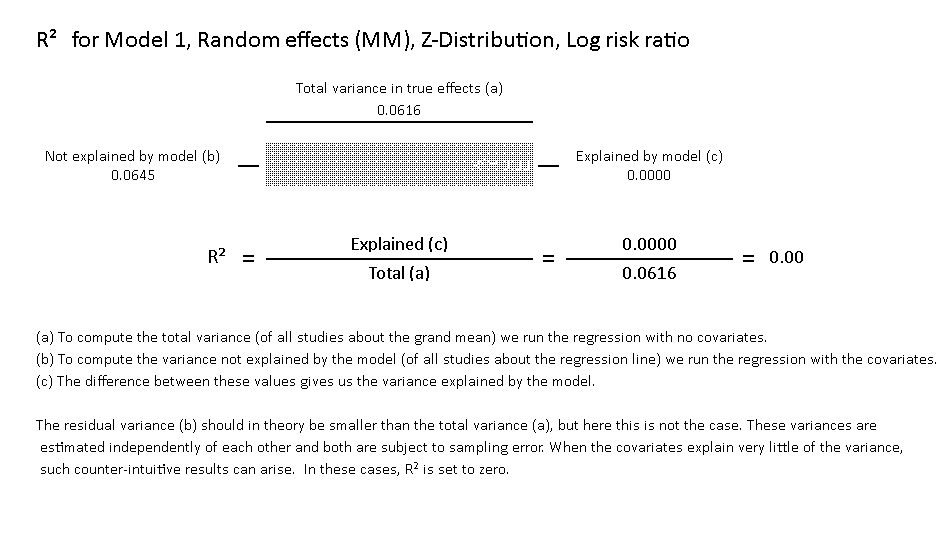


**Symptomatic intracranial hemorrhage sICH** (SE 0.007 95% CI (-0.01–0.01) P value 0.8)

**Male percentage**

mRS 0-1 (SE 0.0046 95%CI (-0.0057–0.023 P value 0.48)

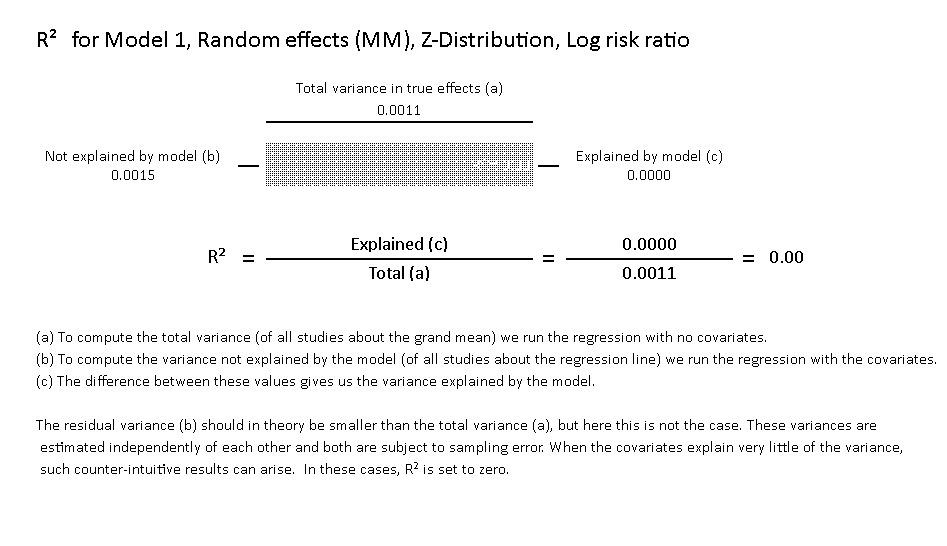


mRS 0-2 at 90 days (SE 0.0048 95%CI (-0.018–0.0009 P value 0.08)

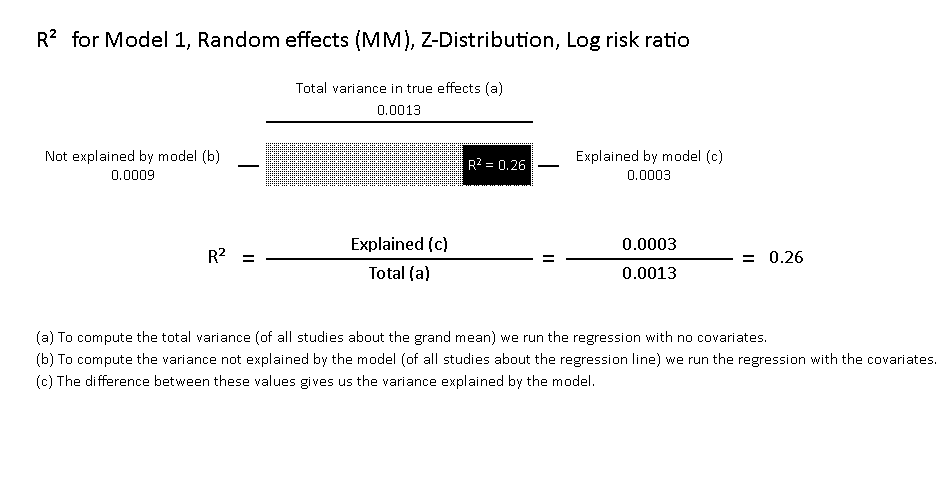


Major neurological improvement (SE 0.007 95%CI (-0.01–0.02 P value 0.8)

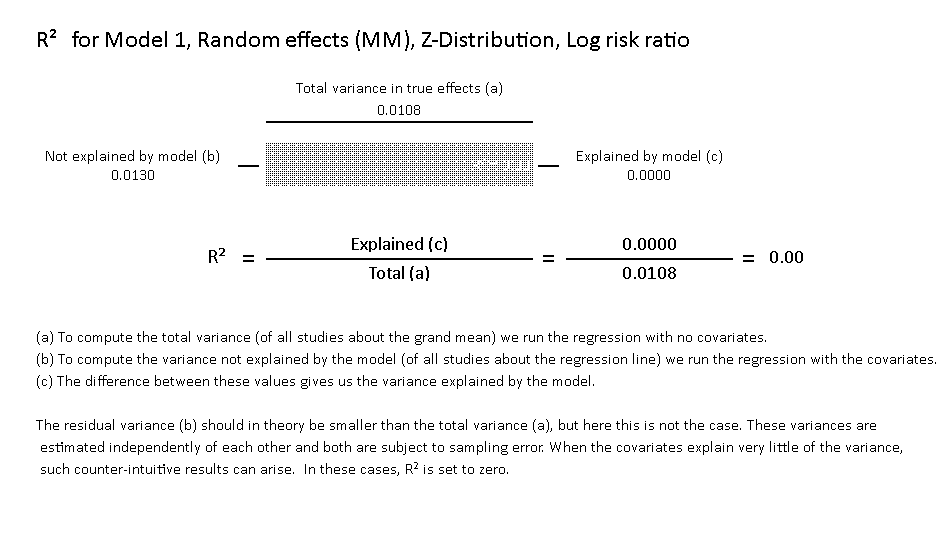


Death (SE 0.02 95%CI (-0.03–0.05 P value 0.7)

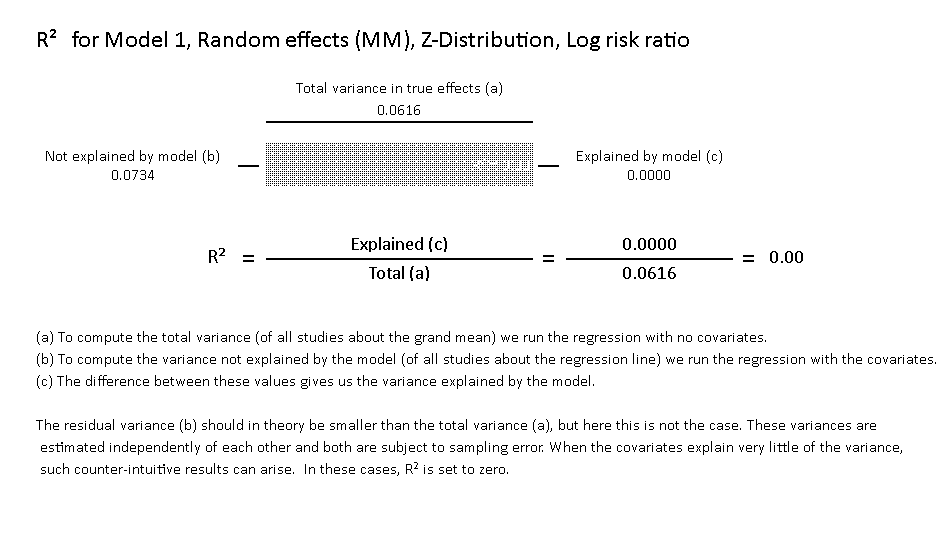


Symptomatic intracranial hemorrhage sICH (SE 0.02 95%CI (-0.04–0.04 P value 0.96)

**hypertension percentage**

mRS 0-1 (SE 0.0027 95%CI (-0.0035–0.0072 P value 0.5)

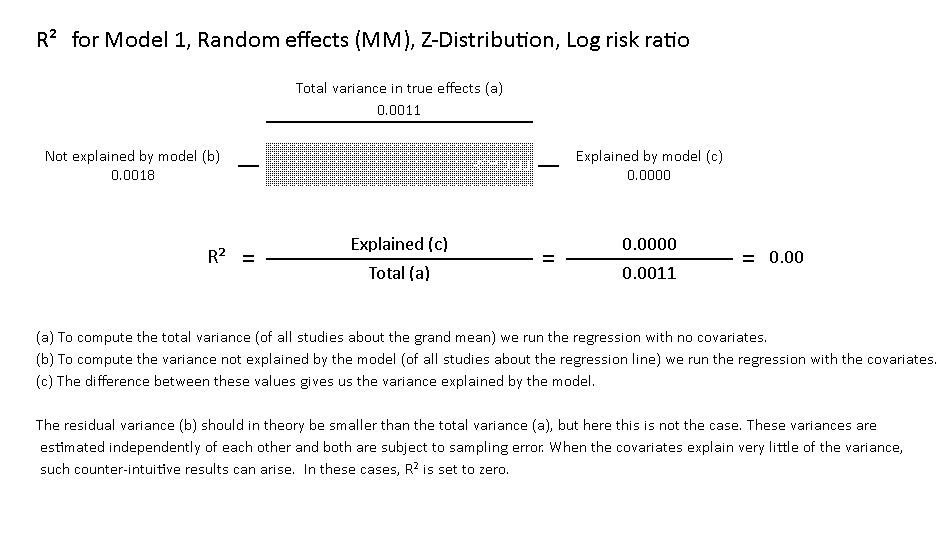


Major neurological improvement (SE 0.06 95%CI (-0.01–0.012 P value 0.85)

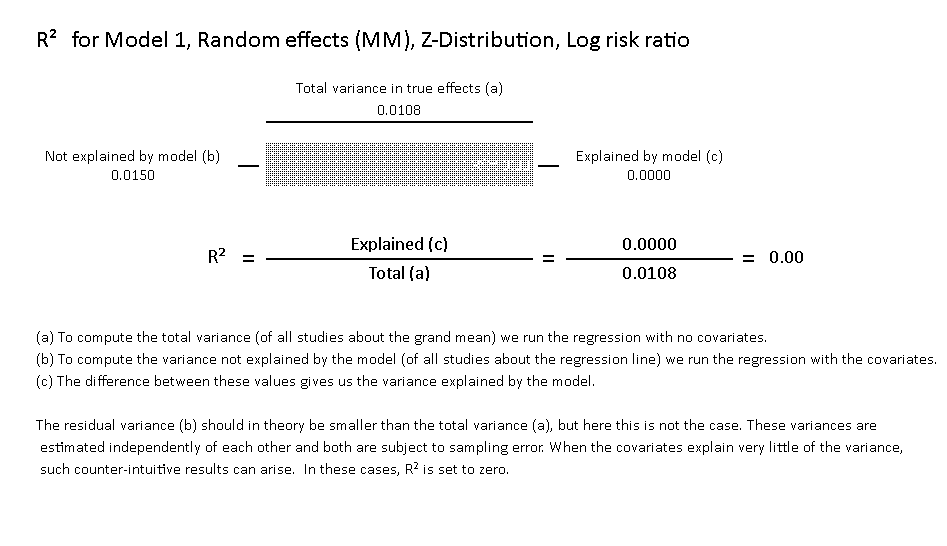


Death (SE 0.01 95%CI (-0.04–0.02 P value 0.4)

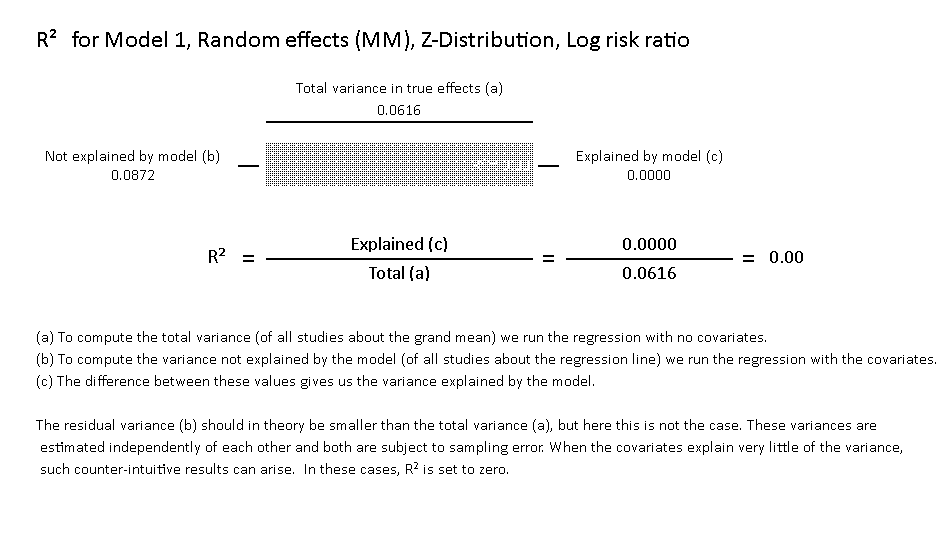


Symptomatic intracranial hemorrhage sICH (SE 0.02 95%CI (-0.02–0.03) P value 0.7)
